# Supplementary figures and images for: Coral micro-fragmentation assays for optimizing active reef restoration efforts
Source: PeerJ. 2022 Jul 18;10:e13653. doi: 10.7717/peerj.13653 (PMC9302430; doi:10.7717/peerj.13653)

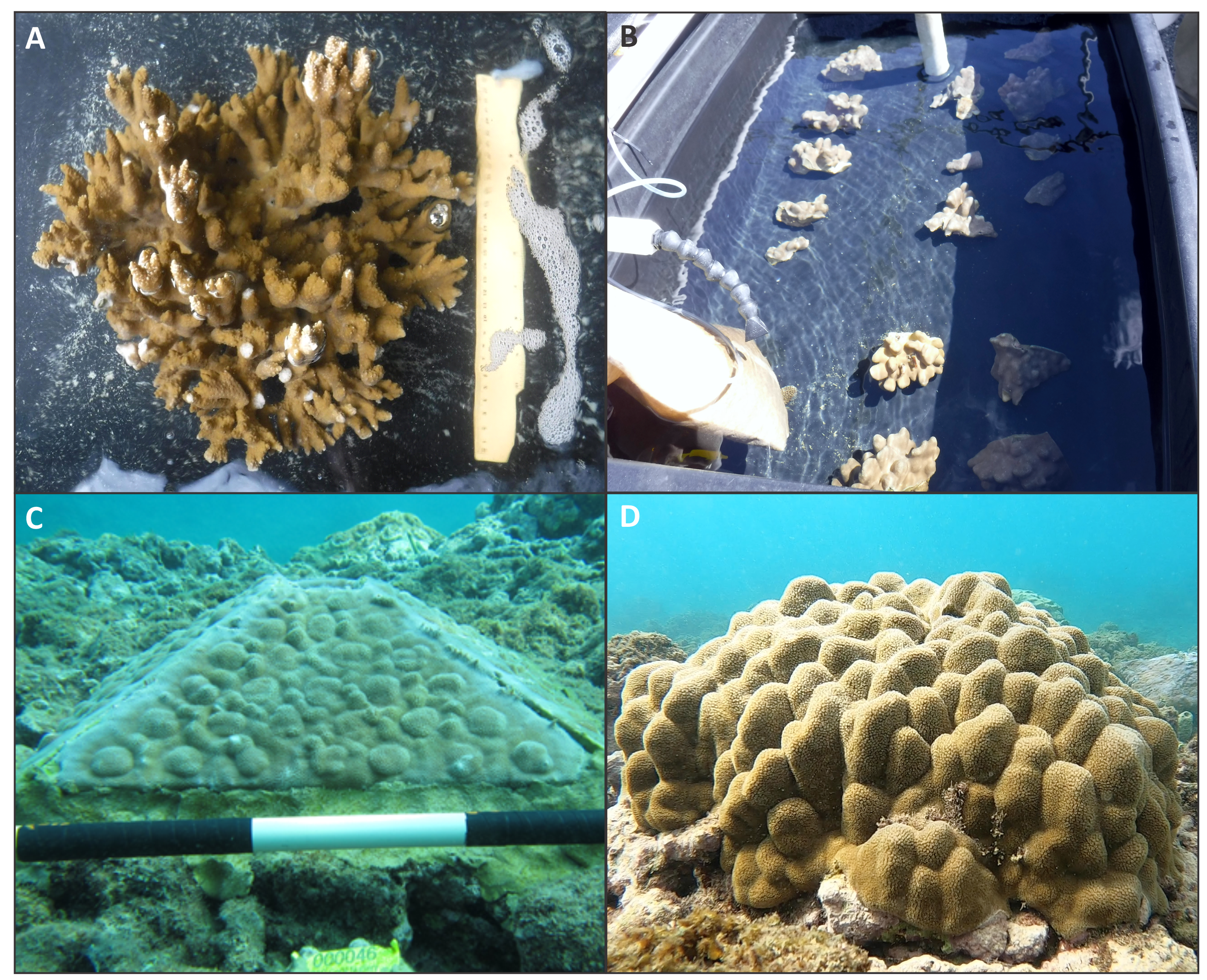

Supplement: Supplemental Information 1 — Images of (A) one of the three Montipora capitata parent colonies from the in-situ nursery (located at the Hawai'i Institute of Marine Biology) and (B) quarantining Porites compressa genotypes 1, 2, and 3 in the ex-situ nursery tanks at Ānuenue Fisheries Research Center (AFRC) Hawai'i Coral Restoration Nursery (HCRN) on Oʻahu, Hawai'i (C) example of a HCRN fully-grown 42 cm pyramid structure with Porites evermanni 1 month after outplanting and (D) the same colony two and half years later (not used in this experiment). [file peerj-10-13653-s001.png]

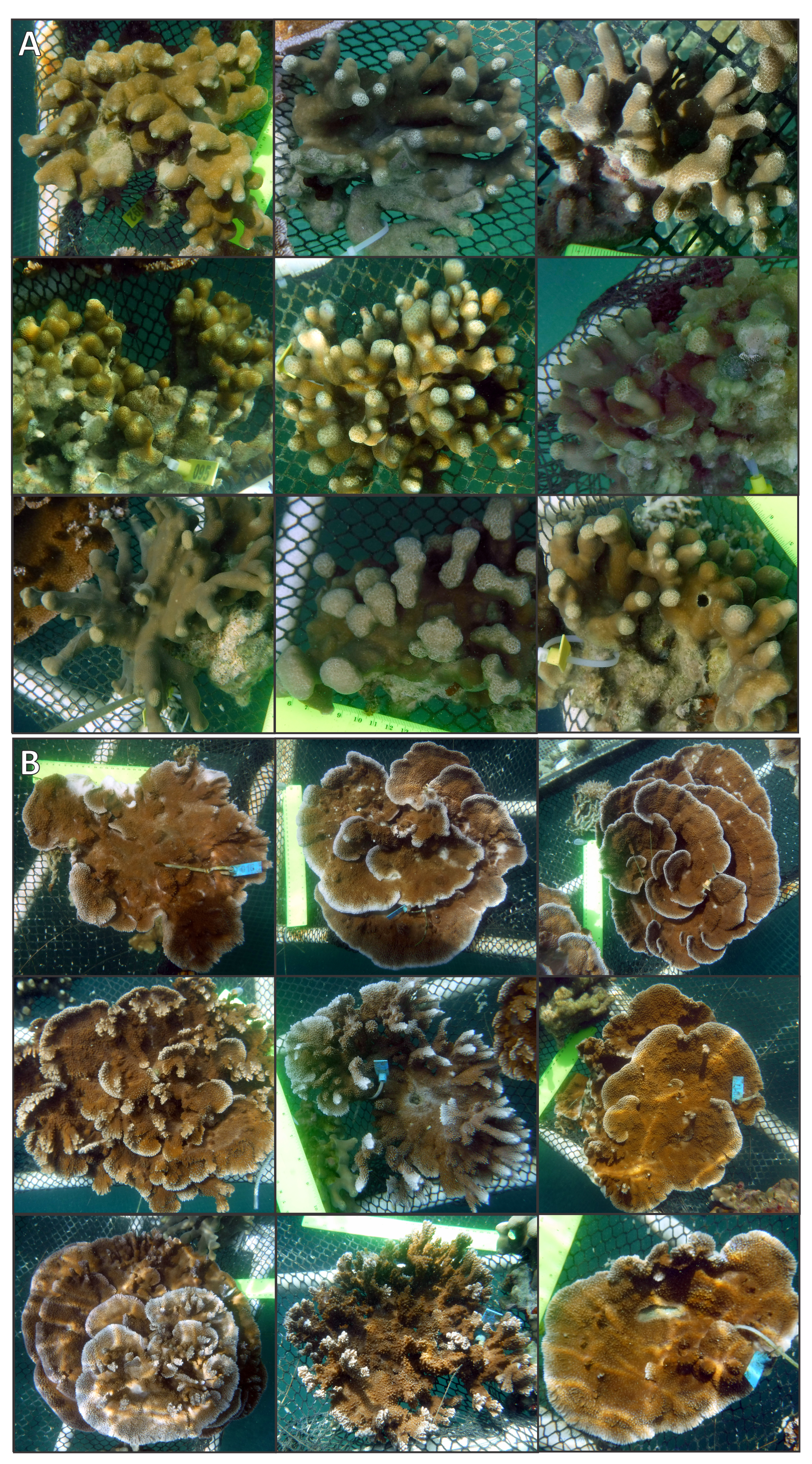

Supplement: Supplemental Information 6 — The block assay (A) nine Porites compressa, and (B) nine Montipora capitata parent/donor colonies located in the in-situ nursery prior to fragmentation. [file peerj-10-13653-s006.png]
