# Supplementary material for: Coral micro-fragmentation assays for optimizing active reef restoration efforts
Source: PeerJ. 2022 Jul 18;10:e13653. doi: 10.7717/peerj.13653 (PMC9302430; doi:10.7717/peerj.13653)
Supplement: Supplemental Information 2 — Timeline of coral assay deployment. Phase 1 (0 months) set 1 was outplanted on the reef and set 2 and 3 were placed into the nursery (total = 54 assays per nursery). Phase 2 and 3 provided 4 months (for set 2) and 8 months (for set 3) of growth respectively in the nursery prior to transplantation on the reef. Phase 4 was the final growth measurement representing 12 months of growth since fragmentation at the in-situ nursery outplanting site and 17 months for the ex-situ site. [file peerj-10-13653-s002.pdf]

Phase	Activity	In-situ nursery	Ex-situ nursery
1	Fragment and measure sets 1, 2 & 3 (n=54)<br>Deployment of set 1 (n=18) (direct transplant)	12/10/2018	02/06/2019
2	Deployment of set 2 (n=18) (4 month nursery growth)	04/04/2019	05/28/2019
3	Deployment of set 3 (n=18) (8 month nursery growth)	08/16/2019	10/18/2019
4	Final measurements of sets 1, 2 & 3 (n=54)	12/12/2019	07/08/2020
