# Supplementary material for: Coral micro-fragmentation assays for optimizing active reef restoration efforts
Source: PeerJ. 2022 Jul 18;10:e13653. doi: 10.7717/peerj.13653 (PMC9302430; doi:10.7717/peerj.13653)
Supplement: Supplemental Information 5 — Pyramid assay statistical analyses with Generalized Linear Mixed Models (glmm) examining net growth, and Linear Mixed effects Models (lmm) assessing fragment survivorship based on total time in the nursery and coral fragment size class (fixed effects), while accounting for variation due to parent colony (genotype) and deployment pyramid within colony (random effects) for Montipora capitata, and Porites compressa fragments at the in-situ and ex-situ nurseries. [file peerj-10-13653-s005.docx]

Pyramid_assay_statistical_analyses

Austin Greene

10/02/2020

**Load libraries**

library(dplyr)
library(ggplot2)
library(lme4)
library(car)
library(MuMIn)
library(sjPlot)
library(glmmTMB)

**Set seed**

set.seed(1234)

**Load data**
Note: Using updated data calculated by Ingrid in September 2020.

getwd()

## [1] "C:/Users/iknap/OneDrive/Documents/coral_nursery_paper/Figures/datasheets_and_Rmarkdown_files"

df <- read.csv("C:/Users/iknap/OneDrive/Documents/coral_nursery_paper/Figures/datasheets_and_Rmarkdown_files/pyramid_assay_growth_data.csv", header = TRUE)

# Calculate net growth from area_1 to area_3
df$AbsoluteNetGrowth <- df$area_3-df$area_1
df$AbsoluteNetGrowth3D <- df$X3D_area_3-df$area_1
df$PercentNetGrowth <- ((df$area_3-df$area_1)/df$area_1)*100
df$PercentNetGrowth3D <- ((df$X3D_area_3-df$area_1)/df$area_1)*100

# head(df)

# Split into separate site data as well...
#HIMB = Hawaii Institute of Marine Biology (in-situ nursery)
#AFRC = Anuenue Fisheries Research Center (ex-situ nursery)

df.HIMB <- df %>%
 subset(site=="HIMB")

# head(df.HIMB)

df.AFRC <- df %>%
 subset(site=="AFRC")

# head(df.AFRC)

***Plots***


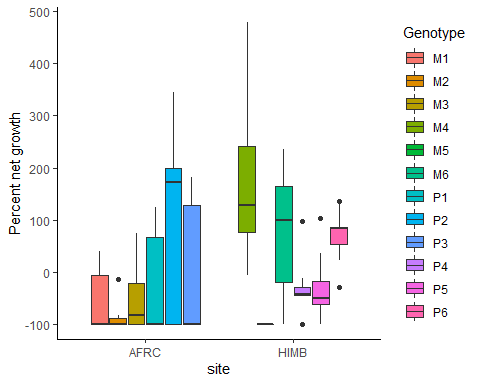


## `geom_smooth()` using method = 'loess' and formula 'y ~ x'

## Warning in simpleLoess(y, x, w, span, degree = degree, parametric =
## parametric, : pseudoinverse used at -1.27

## Warning in simpleLoess(y, x, w, span, degree = degree, parametric =
## parametric, : neighborhood radius 255.27

## Warning in simpleLoess(y, x, w, span, degree = degree, parametric =
## parametric, : reciprocal condition number 1.9387e-016

## Warning in simpleLoess(y, x, w, span, degree = degree, parametric =
## parametric, : There are other near singularities as well. 65163

## Warning in simpleLoess(y, x, w, span, degree = degree, parametric =
## parametric, : pseudoinverse used at -1.27

## Warning in simpleLoess(y, x, w, span, degree = degree, parametric =
## parametric, : neighborhood radius 255.27

## Warning in simpleLoess(y, x, w, span, degree = degree, parametric =
## parametric, : reciprocal condition number 1.9387e-016

## Warning in simpleLoess(y, x, w, span, degree = degree, parametric =
## parametric, : There are other near singularities as well. 65163

## Warning in simpleLoess(y, x, w, span, degree = degree, parametric =
## parametric, : pseudoinverse used at -1.27

## Warning in simpleLoess(y, x, w, span, degree = degree, parametric =
## parametric, : neighborhood radius 255.27

## Warning in simpleLoess(y, x, w, span, degree = degree, parametric =
## parametric, : reciprocal condition number 1.9387e-016

## Warning in simpleLoess(y, x, w, span, degree = degree, parametric =
## parametric, : There are other near singularities as well. 65163

## Warning in simpleLoess(y, x, w, span, degree = degree, parametric =
## parametric, : pseudoinverse used at -1.27

## Warning in simpleLoess(y, x, w, span, degree = degree, parametric =
## parametric, : neighborhood radius 255.27

## Warning in simpleLoess(y, x, w, span, degree = degree, parametric =
## parametric, : reciprocal condition number 1.9387e-016

## Warning in simpleLoess(y, x, w, span, degree = degree, parametric =
## parametric, : There are other near singularities as well. 65163

## Warning in simpleLoess(y, x, w, span, degree = degree, parametric =
## parametric, : pseudoinverse used at -1.27

## Warning in simpleLoess(y, x, w, span, degree = degree, parametric =
## parametric, : neighborhood radius 255.27

## Warning in simpleLoess(y, x, w, span, degree = degree, parametric =
## parametric, : reciprocal condition number 1.9387e-016

## Warning in simpleLoess(y, x, w, span, degree = degree, parametric =
## parametric, : There are other near singularities as well. 65163

## Warning in simpleLoess(y, x, w, span, degree = degree, parametric =
## parametric, : pseudoinverse used at -1.27

## Warning in simpleLoess(y, x, w, span, degree = degree, parametric =
## parametric, : neighborhood radius 255.27

## Warning in simpleLoess(y, x, w, span, degree = degree, parametric =
## parametric, : reciprocal condition number 1.9387e-016

## Warning in simpleLoess(y, x, w, span, degree = degree, parametric =
## parametric, : There are other near singularities as well. 65163

## Warning in simpleLoess(y, x, w, span, degree = degree, parametric =
## parametric, : pseudoinverse used at -1.25

## Warning in simpleLoess(y, x, w, span, degree = degree, parametric =
## parametric, : neighborhood radius 251.25

## Warning in simpleLoess(y, x, w, span, degree = degree, parametric =
## parametric, : reciprocal condition number 4.8893e-017

## Warning in simpleLoess(y, x, w, span, degree = degree, parametric =
## parametric, : There are other near singularities as well. 63127

## Warning in simpleLoess(y, x, w, span, degree = degree, parametric =
## parametric, : pseudoinverse used at -1.25

## Warning in simpleLoess(y, x, w, span, degree = degree, parametric =
## parametric, : neighborhood radius 251.25

## Warning in simpleLoess(y, x, w, span, degree = degree, parametric =
## parametric, : reciprocal condition number 4.8893e-017

## Warning in simpleLoess(y, x, w, span, degree = degree, parametric =
## parametric, : There are other near singularities as well. 63127

## Warning in simpleLoess(y, x, w, span, degree = degree, parametric =
## parametric, : pseudoinverse used at -1.25

## Warning in simpleLoess(y, x, w, span, degree = degree, parametric =
## parametric, : neighborhood radius 251.25

## Warning in simpleLoess(y, x, w, span, degree = degree, parametric =
## parametric, : reciprocal condition number 4.8893e-017

## Warning in simpleLoess(y, x, w, span, degree = degree, parametric =
## parametric, : There are other near singularities as well. 63127

## Warning in simpleLoess(y, x, w, span, degree = degree, parametric =
## parametric, : pseudoinverse used at -1.25

## Warning in simpleLoess(y, x, w, span, degree = degree, parametric =
## parametric, : neighborhood radius 251.25

## Warning in simpleLoess(y, x, w, span, degree = degree, parametric =
## parametric, : reciprocal condition number 4.8893e-017

## Warning in simpleLoess(y, x, w, span, degree = degree, parametric =
## parametric, : There are other near singularities as well. 63127

## Warning in simpleLoess(y, x, w, span, degree = degree, parametric =
## parametric, : pseudoinverse used at -1.25

## Warning in simpleLoess(y, x, w, span, degree = degree, parametric =
## parametric, : neighborhood radius 251.25

## Warning in simpleLoess(y, x, w, span, degree = degree, parametric =
## parametric, : reciprocal condition number 4.8893e-017

## Warning in simpleLoess(y, x, w, span, degree = degree, parametric =
## parametric, : There are other near singularities as well. 63127

## Warning in simpleLoess(y, x, w, span, degree = degree, parametric =
## parametric, : pseudoinverse used at -1.25

## Warning in simpleLoess(y, x, w, span, degree = degree, parametric =
## parametric, : neighborhood radius 251.25

## Warning in simpleLoess(y, x, w, span, degree = degree, parametric =
## parametric, : reciprocal condition number 4.8893e-017

## Warning in simpleLoess(y, x, w, span, degree = degree, parametric =
## parametric, : There are other near singularities as well. 63127

## `geom_smooth()` using method = 'loess' and formula 'y ~ x'

## Warning in simpleLoess(y, x, w, span, degree = degree, parametric =
## parametric, : pseudoinverse used at -1.27

## Warning in simpleLoess(y, x, w, span, degree = degree, parametric =
## parametric, : neighborhood radius 255.27

## Warning in simpleLoess(y, x, w, span, degree = degree, parametric =
## parametric, : reciprocal condition number 4.2524e-016

## Warning in simpleLoess(y, x, w, span, degree = degree, parametric =
## parametric, : There are other near singularities as well. 65163

## Warning in predLoess(object$y, object$x, newx = if
## (is.null(newdata)) object$x else if (is.data.frame(newdata))
## as.matrix(model.frame(delete.response(terms(object)), : pseudoinverse used at
## -1.27

## Warning in predLoess(object$y, object$x, newx = if
## (is.null(newdata)) object$x else if (is.data.frame(newdata))
## as.matrix(model.frame(delete.response(terms(object)), : neighborhood radius
## 255.27

## Warning in predLoess(object$y, object$x, newx = if
## (is.null(newdata)) object$x else if (is.data.frame(newdata))
## as.matrix(model.frame(delete.response(terms(object)), : reciprocal condition
## number 4.2524e-016

## Warning in predLoess(object$y, object$x, newx = if
## (is.null(newdata)) object$x else if (is.data.frame(newdata))
## as.matrix(model.frame(delete.response(terms(object)), : There are other near
## singularities as well. 65163

## Warning in simpleLoess(y, x, w, span, degree = degree, parametric =
## parametric, : pseudoinverse used at -1.25

## Warning in simpleLoess(y, x, w, span, degree = degree, parametric =
## parametric, : neighborhood radius 251.25

## Warning in simpleLoess(y, x, w, span, degree = degree, parametric =
## parametric, : reciprocal condition number 5.8483e-016

## Warning in simpleLoess(y, x, w, span, degree = degree, parametric =
## parametric, : There are other near singularities as well. 63127

## Warning in predLoess(object$y, object$x, newx = if
## (is.null(newdata)) object$x else if (is.data.frame(newdata))
## as.matrix(model.frame(delete.response(terms(object)), : pseudoinverse used at
## -1.25

## Warning in predLoess(object$y, object$x, newx = if
## (is.null(newdata)) object$x else if (is.data.frame(newdata))
## as.matrix(model.frame(delete.response(terms(object)), : neighborhood radius
## 251.25

## Warning in predLoess(object$y, object$x, newx = if
## (is.null(newdata)) object$x else if (is.data.frame(newdata))
## as.matrix(model.frame(delete.response(terms(object)), : reciprocal condition
## number 5.8483e-016

## Warning in predLoess(object$y, object$x, newx = if
## (is.null(newdata)) object$x else if (is.data.frame(newdata))
## as.matrix(model.frame(delete.response(terms(object)), : There are other near
## singularities as well. 63127


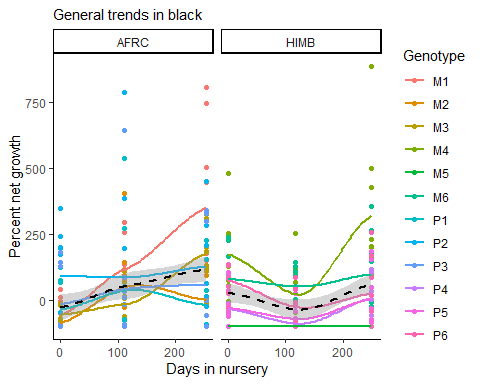


### Testing if percent net growth from T1-T3 was significantly driven by time in a nursery or size of fragment

Running test separately for each site and species, accounting for replicates (pyramids) within each genotype

**MCAP at AFRC**

lm.1.AFRC.MCAP <- lmer(PercentNetGrowth ~ exp_day_outplanted*size + (1|genotype/pyramid) , data = subset(df.AFRC,species=="MCAP"))

summary(lm.1.AFRC.MCAP)

## Linear mixed model fit by REML ['lmerMod']
## Formula: PercentNetGrowth ~ exp_day_outplanted * size + (1 | genotype/pyramid)
## Data: subset(df.AFRC, species == "MCAP")
##
## REML criterion at convergence: 1002.4
##
## Scaled residuals:
## Min 1Q Median 3Q Max
## -2.0421 -0.5336 -0.0771 0.3621 3.3455
##
## Random effects:
## Groups Name Variance Std.Dev.
## pyramid:genotype (Intercept) 3219 56.73
## genotype (Intercept) 4828 69.48
## Residual 16112 126.93
## Number of obs: 81, groups: pyramid:genotype, 27; genotype, 3
##
## Fixed effects:
## Estimate Std. Error t value
## (Intercept) -38.0312 57.4953 -0.661
## exp_day_outplanted 0.5114 0.2574 1.987
## sizeMedium -15.9257 53.1771 -0.299
## sizeSmall -60.2118 53.1771 -1.132
## exp_day_outplanted:sizeMedium 0.4145 0.3323 1.248
## exp_day_outplanted:sizeSmall 0.8947 0.3323 2.693
##
## Correlation of Fixed Effects:
## (Intr) exp_d_ sizMdm szSmll ex__:M
## exp_dy_tpln -0.545
## sizeMedium -0.462 0.491
## sizeSmall -0.462 0.491 0.500
## exp_dy_tp:M 0.352 -0.646 -0.760 -0.380
## exp_dy_tp:S 0.352 -0.646 -0.380 -0.760 0.500

Anova(lm.1.AFRC.MCAP, type = 3)

## Analysis of Deviance Table (Type III Wald chisquare tests)
##
## Response: PercentNetGrowth
## Chisq Df Pr(>Chisq)
## (Intercept) 0.4375 1 0.50831
## exp_day_outplanted 3.9494 1 0.04689 *
## size 1.3769 2 0.50236
## exp_day_outplanted:size 7.2633 2 0.02647 *
## ---
## Signif. codes: 0 '***' 0.001 '**' 0.01 '*' 0.05 '.' 0.1 ' ' 1

Significant effect at AFRC of nursery time, though note the small estimated effect size. Significant interaction between size group and nursery time, though these effects are also small.

**PCOMP at AFRC**

lm.1.AFRC.PCOMP <- lmer(PercentNetGrowth ~ exp_day_outplanted*size + (1|genotype/pyramid) , data = subset(df.AFRC,species=="PCOMP"))

summary(lm.1.AFRC.PCOMP)

## Linear mixed model fit by REML ['lmerMod']
## Formula: PercentNetGrowth ~ exp_day_outplanted * size + (1 | genotype/pyramid)
## Data: subset(df.AFRC, species == "PCOMP")
##
## REML criterion at convergence: 1042
##
## Scaled residuals:
## Min 1Q Median 3Q Max
## -1.7479 -0.5378 -0.2140 0.3742 2.9200
##
## Random effects:
## Groups Name Variance Std.Dev.
## pyramid:genotype (Intercept) 16956 130.21
## genotype (Intercept) 282 16.79
## Residual 22571 150.24
## Number of obs: 81, groups: pyramid:genotype, 27; genotype, 3
##
## Fixed effects:
## Estimate Std. Error t value
## (Intercept) 17.06226 59.68806 0.286
## exp_day_outplanted 0.17259 0.36801 0.469
## sizeMedium 45.55385 62.93990 0.724
## sizeSmall -34.86906 62.93990 -0.554
## exp_day_outplanted:sizeMedium -0.02402 0.39328 -0.061
## exp_day_outplanted:sizeSmall -0.02396 0.39328 -0.061
##
## Correlation of Fixed Effects:
## (Intr) exp_d_ sizMdm szSmll ex__:M
## exp_dy_tpln -0.750
## sizeMedium -0.527 0.406
## sizeSmall -0.527 0.406 0.500
## exp_dy_tp:M 0.401 -0.534 -0.760 -0.380
## exp_dy_tp:S 0.401 -0.534 -0.380 -0.760 0.500

Anova(lm.1.AFRC.PCOMP, type = 3)

## Analysis of Deviance Table (Type III Wald chisquare tests)
##
## Response: PercentNetGrowth
## Chisq Df Pr(>Chisq)
## (Intercept) 0.0817 1 0.7750
## exp_day_outplanted 0.2199 1 0.6391
## size 1.6423 2 0.4399
## exp_day_outplanted:size 0.0050 2 0.9975

No significant effects anywhere for PCOMP at AFRC.

Lets confirm how the data looks with a boxplot containing both size class and nursery residence time for each species and size class.

ggplot(data=df.AFRC) +
 geom_boxplot(aes(x=size, y=PercentNetGrowth)) +
 facet_wrap(vars(species,exp_day_outplanted)) +
 labs(x="Fragment size class", y="Percent net growth") +
 theme_classic()


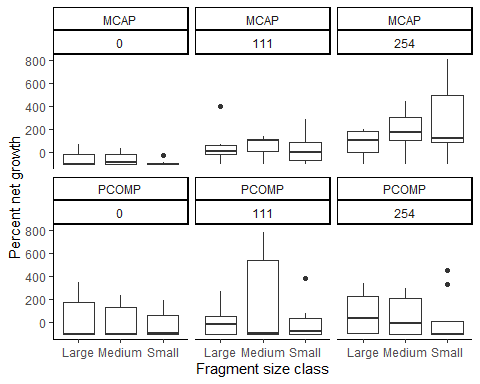


We can see clearly here that size classes of PCOMP at AFRC do not appear to significantly differ with the duration they are kept in a nursery. On the other hand, MCAP fragments clearly respond to nursery time differently depending on what size class they are. These plots support our model findings above.

**MCAP at HIMB**
NOTE: I already tested if the model below differs using 3D size data. It does not.

lm.1.HIMB.MCAP <- lmer(PercentNetGrowth ~ exp_day_outplanted*size + (1|genotype/pyramid) , data = subset(df.HIMB,species=="MCAP"))

summary(lm.1.HIMB.MCAP)

## Linear mixed model fit by REML ['lmerMod']
## Formula: PercentNetGrowth ~ exp_day_outplanted * size + (1 | genotype/pyramid)
## Data: subset(df.HIMB, species == "MCAP")
##
## REML criterion at convergence: 987.8
##
## Scaled residuals:
## Min 1Q Median 3Q Max
## -1.9575 -0.3430 -0.0362 0.1984 3.7569
##
## Random effects:
## Groups Name Variance Std.Dev.
## pyramid:genotype (Intercept) 12527 111.93
## genotype (Intercept) 17148 130.95
## Residual 8779 93.69
## Number of obs: 81, groups: pyramid:genotype, 27; genotype, 3
##
## Fixed effects:
## Estimate Std. Error t value
## (Intercept) 12.37840 87.35302 0.142
## exp_day_outplanted 0.26473 0.27500 0.963
## sizeMedium 13.72079 39.72159 0.345
## sizeSmall 8.62209 39.72159 0.217
## exp_day_outplanted:sizeMedium -0.02171 0.24964 -0.087
## exp_day_outplanted:sizeSmall -0.06631 0.24964 -0.266
##
## Correlation of Fixed Effects:
## (Intr) exp_d_ sizMdm szSmll ex__:M
## exp_dy_tpln -0.384
## sizeMedium -0.227 0.348
## sizeSmall -0.227 0.348 0.500
## exp_dy_tp:M 0.174 -0.454 -0.767 -0.383
## exp_dy_tp:S 0.174 -0.454 -0.383 -0.767 0.500

Anova(lm.1.HIMB.MCAP, type = 3)

## Analysis of Deviance Table (Type III Wald chisquare tests)
##
## Response: PercentNetGrowth
## Chisq Df Pr(>Chisq)
## (Intercept) 0.0201 1 0.8873
## exp_day_outplanted 0.9267 1 0.3357
## size 0.1219 2 0.9409
## exp_day_outplanted:size 0.0734 2 0.9640

No significant effects anywhere for MCAP at HIMB.

**PCOMP at HIMB**
NOTE: I already tested if the model below differs using 3D size data. It does not.

lm.1.HIMB.PCOMP <- lmer(PercentNetGrowth ~ exp_day_outplanted*size + (1|genotype/pyramid) , data = subset(df.HIMB,species=="PCOMP"))

summary(lm.1.HIMB.PCOMP)

## Linear mixed model fit by REML ['lmerMod']
## Formula: PercentNetGrowth ~ exp_day_outplanted * size + (1 | genotype/pyramid)
## Data: subset(df.HIMB, species == "PCOMP")
##
## REML criterion at convergence: 909.8
##
## Scaled residuals:
## Min 1Q Median 3Q Max
## -1.54275 -0.47754 -0.05318 0.33967 2.84902
##
## Random effects:
## Groups Name Variance Std.Dev.
## pyramid:genotype (Intercept) 2554.4 50.54
## genotype (Intercept) 775.3 27.84
## Residual 3901.2 62.46
## Number of obs: 81, groups: pyramid:genotype, 27; genotype, 3
##
## Fixed effects:
## Estimate Std. Error t value
## (Intercept) -40.18285 28.95827 -1.388
## exp_day_outplanted 0.01941 0.15137 0.128
## sizeMedium 31.12291 26.47960 1.175
## sizeSmall 20.58735 26.47960 0.777
## exp_day_outplanted:sizeMedium 0.19906 0.16641 1.196
## exp_day_outplanted:sizeSmall -0.11896 0.16641 -0.715
##
## Correlation of Fixed Effects:
## (Intr) exp_d_ sizMdm szSmll ex__:M
## exp_dy_tpln -0.638
## sizeMedium -0.457 0.421
## sizeSmall -0.457 0.421 0.500
## exp_dy_tp:M 0.351 -0.550 -0.767 -0.383
## exp_dy_tp:S 0.351 -0.550 -0.383 -0.767 0.500

Anova(lm.1.HIMB.PCOMP, type = 3)

## Analysis of Deviance Table (Type III Wald chisquare tests)
##
## Response: PercentNetGrowth
## Chisq Df Pr(>Chisq)
## (Intercept) 1.9255 1 0.1653
## exp_day_outplanted 0.0164 1 0.8980
## size 1.4295 2 0.4893
## exp_day_outplanted:size 3.7292 2 0.1550

Of anything for PCOMP at HIMB

Let’s confirm these size results with a boxplot…

ggplot(data=df.HIMB) +
 geom_boxplot(aes(x=size, y=PercentNetGrowth)) +
 facet_wrap(vars(species,exp_day_outplanted)) +
 labs(x="Fragment size class", y="Percent net growth") +
 theme_classic()


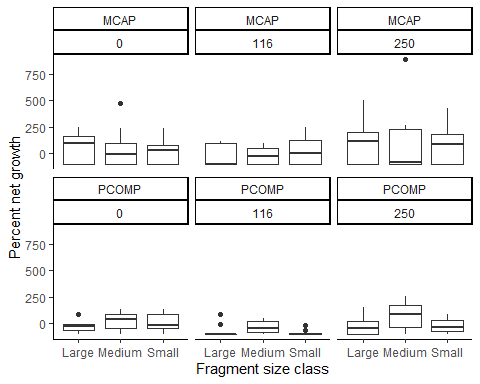


The plot above suggests that medium size fragments for PCOMP at HIMB might be at a slight advantage, but this is not statistically significant.

**These findings suggest a couple things**

- Nursery time at AFRC benefits MCAP, but does not benefit PCOMP. This suggests that conditions at AFRC are likely sub-optimal for PCOMP.
- At AFRC, how long you retain a fragment in a nursery environment needs to be dependent on fragment size. In other words, no one fragment size is ideal to outplant across all nursery durations.
- At HIMB, no amount of nursery time significantly improves the growth of a fragment, regardless of size. These results suggest the in-situ HIMB nursery may be sub-optimal in general and has no effect versus direct outplanting at time of fragmentation.

**Note 1**: All of these results assume that the reef environments fragments are deployed onto at HIMB or AFRC are comparable. They are not. The growth of a fragment is a product of both nursery conditions, size, AND where it is outplanted to.

### Survivorship

Next we’ll address the probability of a fragment surviving after outplanting, and if it is significantly impacted by nursery location, species, or time in nursery. Note that these tests are being done at the fragment scale.

**Importing fragment-scale survivorship data**

getwd()

## [1] "C:/Users/iknap/OneDrive/Documents/coral_nursery_paper/Figures/datasheets_and_Rmarkdown_files"

df.survivorship <- read.csv("C:/Users/iknap/OneDrive/Documents/coral_nursery_paper/Figures/datasheets_and_Rmarkdown_files/pyramid_assay_survivorship_data.csv", header = TRUE)

# Split into separate site data as well...
df.survivorship.HIMB <- df.survivorship %>%
 subset(site=="HIMB")

# head(df.HIMB)

df.survivorship.AFRC <- df.survivorship %>%
 subset(site=="AFRC")

# head(df.AFRC)

# Column "alive_1" is whether or not a fragment was alive at time of outplanting
# Column "alive_2" is whether or not a fragment was alive at the end of the experiment
# Column "exp_day_outplanted" is how long a fragment stayed in nursery

# Note that each row is a single fragment. 1188 rows

# Subset to only fragments that survived to outplanting
df.survivorship.outplantalive <- subset(df.survivorship, alive_1 > 0)

# Split into separate site data as well...
df.survivorship.outplantalive.HIMB <- df.survivorship.outplantalive %>%
 subset(site=="HIMB")

# head(df.HIMB)

df.survivorship.outplantalive.AFRC <- df.survivorship.outplantalive %>%
 subset(site=="AFRC")

# nrow(df.survivorship) #1188 in df of all frags
# nrow(df.survivorship.outplantalive) #1062 surviving until outplanting

We’ll assess the probability of a fragment dying as a binomial response, with a glmm. I don’t think we need to adjust the model structure from before, just now it is a glmm because of the binomial response. We still have pyramid a fragment belongs to nested within genotype. We could put “face” as a random group, but in this glmm of n choose k, n within the size group/genotype/pyramid combination IS the number of fragments on the face. We’ll see in the model summary if those groups are correctly distinguished.

NOTE: Tested including a third level of random effects, no clear improvement in model. Removed to keep it simple.

NOTE: The response here, “alive_2” is 1 = surviving and 0 = fragment dead.

**Binomial model of fragment mortality, HIMB MCAP**

# In the model call we need to scale exp_day_outplanted or we get an eigenvalue warning. Doesn't change results.

glm1.survival.HIMB.MCAP <- glmer(alive_2 ~
 scale(exp_day_outplanted)*size + (1|genotype/pyramid),
 data = subset(df.survivorship.outplantalive.HIMB, species=="MCAP"),
 family = binomial,
 control = glmerControl(optimizer = "nloptwrap"))

## Warning in checkConv(attr(opt, "derivs"), opt$par, ctrl = control$checkConv, :
## Model failed to converge with max|grad| = 0.0462277 (tol = 0.002, component 1)

## Warning in checkConv(attr(opt, "derivs"), opt$par, ctrl = control$checkConv, : Model is nearly unidentifiable: very large eigenvalue
## - Rescale variables?

summary(glm1.survival.HIMB.MCAP)

## Generalized linear mixed model fit by maximum likelihood (Laplace
## Approximation) [glmerMod]
## Family: binomial ( logit )
## Formula: alive_2 ~ scale(exp_day_outplanted) * size + (1 | genotype/pyramid)
## Data: subset(df.survivorship.outplantalive.HIMB, species == "MCAP")
## Control: glmerControl(optimizer = "nloptwrap")
##
## AIC BIC logLik deviance df.resid
## 235.1 263.0 -109.6 219.1 232
##
## Scaled residuals:
## Min 1Q Median 3Q Max
## -2.8959 -0.1026 0.2603 0.5880 1.2133
##
## Random effects:
## Groups Name Variance Std.Dev.
## pyramid:genotype (Intercept) 0.4737 0.6883
## genotype (Intercept) 12.8214 3.5807
## Number of obs: 240, groups: pyramid:genotype, 26; genotype, 3
##
## Fixed effects:
## Estimate Std. Error z value Pr(>|z|)
## (Intercept) -0.8624864 0.0018580 -464.205 <2e-16
## scale(exp_day_outplanted) 0.6490476 0.0017009 381.584 <2e-16
## sizeMedium -0.5032292 0.3982818 -1.264 0.206
## sizeSmall -0.9660172 0.0017951 -538.154 <2e-16
## scale(exp_day_outplanted):sizeMedium -0.1174934 0.0017951 -65.454 <2e-16
## scale(exp_day_outplanted):sizeSmall 0.0003121 0.0017629 0.177 0.859
##
## (Intercept) ***
## scale(exp_day_outplanted) ***
## sizeMedium
## sizeSmall ***
## scale(exp_day_outplanted):sizeMedium ***
## scale(exp_day_outplanted):sizeSmall
## ---
## Signif. codes: 0 '***' 0.001 '**' 0.01 '*' 0.05 '.' 0.1 ' ' 1
##
## Correlation of Fixed Effects:
## (Intr) sc(__) sizMdm szSmll s(__):M
## scl(xp_dy_) 0.000
## sizeMedium -0.002 0.003
## sizeSmall -0.207 0.000 0.002
## scl(xp__):M 0.207 0.000 0.003 0.196
## scl(xp__):S 0.263 0.000 0.002 -0.055 0.055
## optimizer (nloptwrap) convergence code: 0 (OK)
## Model failed to converge with max|grad| = 0.0462277 (tol = 0.002, component 1)
## Model is nearly unidentifiable: very large eigenvalue
## - Rescale variables?

# R2
r.squaredGLMM(glm1.survival.HIMB.MCAP)

## Warning: 'r.squaredGLMM' now calculates a revised statistic. See the help page.

## Warning: The null model is correct only if all variables used by the original
## model remain unchanged.

## R2m R2c
## theoretical 0.02828847 0.8072468
## delta 0.02711334 0.7737130

# Convergence
relgrad <- with(glm1.survival.HIMB.MCAP@optinfo$derivs,solve(Hessian,gradient))
max(abs(relgrad))

## [1] 0.002074758

#VIF
vif(glm1.survival.HIMB.MCAP)

## GVIF Df GVIF^(1/(2*Df))
## scale(exp_day_outplanted) 1.000009 1 1.000004
## size 1.044767 2 1.011009
## scale(exp_day_outplanted):size 1.044758 2 1.011006

This model in unstable with borderline convergence. Convergence improves if we remove the random effect for pyramid, but variance inflation increases. It could simply be that there isn’t enough variation in the data? Model convergence improves slightly with size group within pyramid within genotype but not really in a meaningful way. In all of these cases no effects are significant. Let’s stick with the original model spec for now and check the other coral groupings for effects. First, review the diagnostic plots for this model…

plot_model(glm1.survival.HIMB.MCAP, type = "std", show.intercept = TRUE, show.p = TRUE) +
 theme_classic() +
 labs(title = "Estimated effect-size on fragment P(survival) at 367 days")

## Warning in checkConv(attr(opt, "derivs"), opt$par, ctrl = control$checkConv, :
## unable to evaluate scaled gradient

## Warning in checkConv(attr(opt, "derivs"), opt$par, ctrl = control$checkConv, :
## Model failed to converge: degenerate Hessian with 1 negative eigenvalues

## Argument 'df_method' is deprecated. Please use 'ci_method' instead.

## Warning in vcov.merMod(object, use.hessian = use.hessian): variance-covariance matrix computed from finite-difference Hessian is
## not positive definite or contains NA values: falling back to var-cov estimated from RX

## Warning in vcov.merMod(object, correlation = correlation, sigm = sig): variance-covariance matrix computed from finite-difference Hessian is
## not positive definite or contains NA values: falling back to var-cov estimated from RX

## Warning in vcov.merMod(object, use.hessian = use.hessian): variance-covariance matrix computed from finite-difference Hessian is
## not positive definite or contains NA values: falling back to var-cov estimated from RX

## Warning in vcov.merMod(object, correlation = correlation, sigm = sig): variance-covariance matrix computed from finite-difference Hessian is
## not positive definite or contains NA values: falling back to var-cov estimated from RX

## Warning in vcov.merMod(object, use.hessian = use.hessian): variance-covariance matrix computed from finite-difference Hessian is
## not positive definite or contains NA values: falling back to var-cov estimated from RX

## Warning in vcov.merMod(object, correlation = correlation, sigm = sig): variance-covariance matrix computed from finite-difference Hessian is
## not positive definite or contains NA values: falling back to var-cov estimated from RX

## Warning in vcov.merMod(object, use.hessian = use.hessian): variance-covariance matrix computed from finite-difference Hessian is
## not positive definite or contains NA values: falling back to var-cov estimated from RX

## Warning in vcov.merMod(object, correlation = correlation, sigm = sig): variance-covariance matrix computed from finite-difference Hessian is
## not positive definite or contains NA values: falling back to var-cov estimated from RX


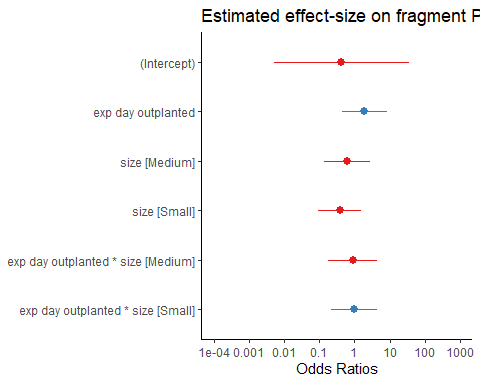


plot_model(glm1.survival.HIMB.MCAP, type = "diag")

## $`pyramid:genotype`

## `geom_smooth()` using formula 'y ~ x'


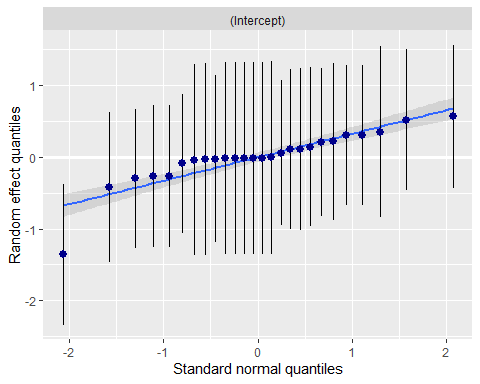


##
## $genotype

## `geom_smooth()` using formula 'y ~ x'


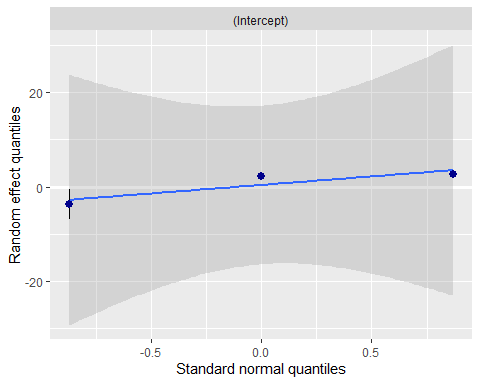


The diagnostic plots demonstrate the problem with the HIMB MCAP data, data within pyramids are highly variable and noisy compared to other species or sites. Almost no variation attributed to genotypes, consistent with model summary.

**Plotting marginal predicted effects, where non-focal variables are held constant**

plot_model(glm1.survival.HIMB.MCAP, type = "eff", title = "Predicted probability of fragment survival at Day 367", axis.title = c("P(Fragment surviving at Day 367)"))

## Package `effects` is not available, but needed for `ggeffect()`. Either install package `effects`, or use `ggpredict()`. Calling `ggpredict()` now.FALSE

## $exp_day_outplanted


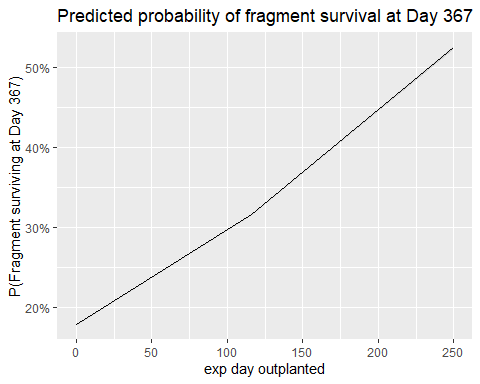


##
## $size


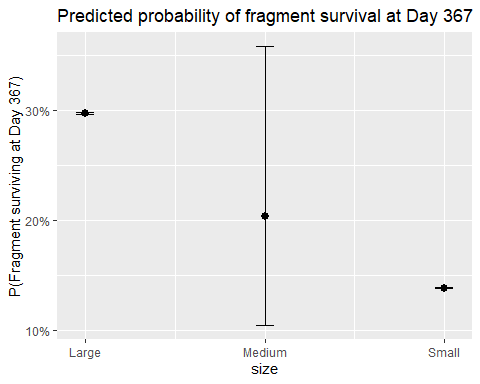


You can see in these marginal plots that predictions based on marginal effects show almost no difference between fragments of different sizes or fragments of different nursery durations. Consistent with model summary.

**Binomial model of fragment mortality, HIMB PCOMP**

glm1.survival.HIMB.PCOMP <- glmer(alive_2 ~
 scale(exp_day_outplanted)*size + (1|genotype/pyramid),
 data = subset(df.survivorship.outplantalive.HIMB, species=="PCOMP"),
 family = binomial,
 control = glmerControl(optimizer = "nloptwrap"))

summary(glm1.survival.HIMB.PCOMP)

## Generalized linear mixed model fit by maximum likelihood (Laplace
## Approximation) [glmerMod]
## Family: binomial ( logit )
## Formula: alive_2 ~ scale(exp_day_outplanted) * size + (1 | genotype/pyramid)
## Data: subset(df.survivorship.outplantalive.HIMB, species == "PCOMP")
## Control: glmerControl(optimizer = "nloptwrap")
##
## AIC BIC logLik deviance df.resid
## 272.8 300.4 -128.4 256.8 224
##
## Scaled residuals:
## Min 1Q Median 3Q Max
## -3.3879 -0.5654 0.1041 0.5423 2.3050
##
## Random effects:
## Groups Name Variance Std.Dev.
## pyramid:genotype (Intercept) 1.4892 1.2203
## genotype (Intercept) 0.8591 0.9269
## Number of obs: 232, groups: pyramid:genotype, 26; genotype, 3
##
## Fixed effects:
## Estimate Std. Error z value Pr(>|z|)
## (Intercept) 0.4544 0.7670 0.592 0.5536
## scale(exp_day_outplanted) -0.1104 0.5674 -0.195 0.8457
## sizeMedium 0.8714 0.6083 1.433 0.1520
## sizeSmall -0.8479 0.5369 -1.579 0.1142
## scale(exp_day_outplanted):sizeMedium 1.1958 0.6491 1.842 0.0654 .
## scale(exp_day_outplanted):sizeSmall 0.2643 0.5459 0.484 0.6283
## ---
## Signif. codes: 0 '***' 0.001 '**' 0.01 '*' 0.05 '.' 0.1 ' ' 1
##
## Correlation of Fixed Effects:
## (Intr) sc(__) sizMdm szSmll s(__):M
## scl(xp_dy_) -0.057
## sizeMedium -0.508 0.056
## sizeSmall -0.586 0.062 0.722
## scl(xp__):M 0.044 -0.694 0.088 -0.075
## scl(xp__):S 0.049 -0.831 -0.058 -0.064 0.726

# R2
r.squaredGLMM(glm1.survival.HIMB.PCOMP)

## Warning: The null model is correct only if all variables used by the original
## model remain unchanged.

## R2m R2c
## theoretical 0.1505589 0.5043467
## delta 0.1357580 0.4547662

# Convergence
relgrad <- with(glm1.survival.HIMB.PCOMP@optinfo$derivs,solve(Hessian,gradient))
max(abs(relgrad))

## [1] 0.0003705275

#VIF
vif(glm1.survival.HIMB.PCOMP)

## GVIF Df GVIF^(1/(2*Df))
## scale(exp_day_outplanted) 3.448702 1 1.857068
## size 1.127560 2 1.030469
## scale(exp_day_outplanted):size 3.842808 2 1.400110

Good news, the model here is stable and VIF is good. To me that suggests the specification is acceptable and our problems with MCAP before were probably down to poor data for the question at hand. That said, the story here is the same. No significant effects on the probability that individual fragments die once outplanted.

plot_model(glm1.survival.HIMB.PCOMP, type = "std", show.intercept = TRUE, show.p = TRUE) +
 theme_classic() +
 labs(title = "Estimated effect-size on fragment P(survival) at 367 days")

## Argument 'df_method' is deprecated. Please use 'ci_method' instead.


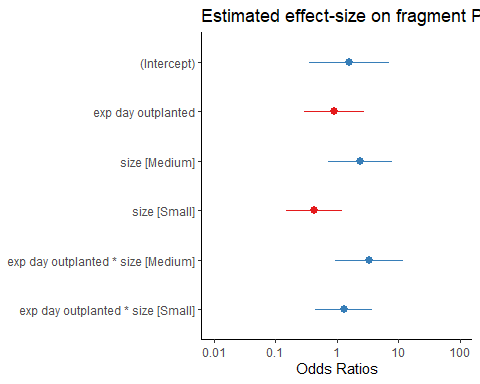


plot_model(glm1.survival.HIMB.PCOMP, type = "diag")

## $`pyramid:genotype`

## `geom_smooth()` using formula 'y ~ x'


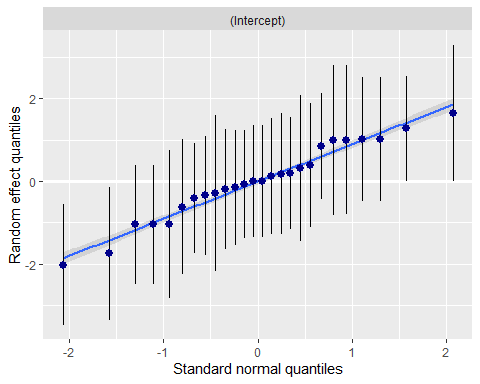


##
## $genotype

## `geom_smooth()` using formula 'y ~ x'


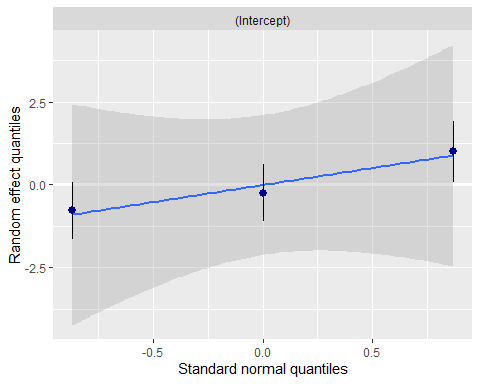


Diagnostic plots provide evidence that genotypes hold very little variation (though more than MCAP at HIMB) and the variation amid pyramids fits our expectation of being normally distributed. That is good news for the model specification.

**Plotting marginal predicted effects, where non-focal variables are held constant**

plot_model(glm1.survival.HIMB.PCOMP, type = "eff", title = "Predicted probability of fragment survival at Day 367", axis.title = c("P(Fragment surviving at Day 367)"))

## Package `effects` is not available, but needed for `ggeffect()`. Either install package `effects`, or use `ggpredict()`. Calling `ggpredict()` now.FALSE

## $exp_day_outplanted


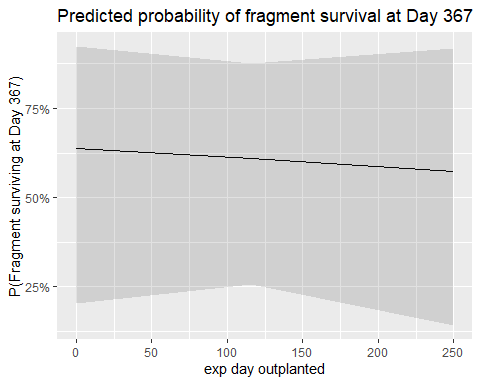


##
## $size


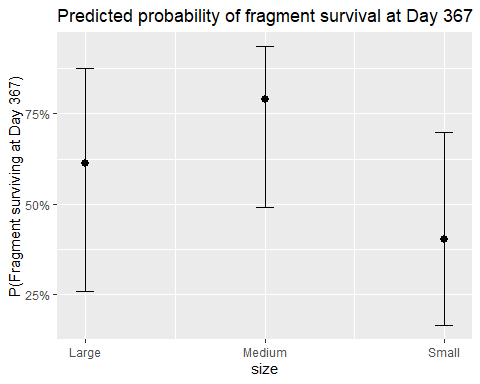


Note the difference in baseline predicted survival for nursery time at HIMB compared to AFRC. Also, note that at HIMB for PCOMP we see increased survivorship for medium fragments. While size group alone was not significant in the model summary, this difference in the marginal plot points toward one component of the significant interaction between medium fragment size and nursery time.

**Binomial model of fragment mortality, AFRC MCAP**

glm1.survival.AFRC.MCAP <- glmer(alive_2 ~
 scale(exp_day_outplanted)*size + (1|genotype/pyramid),
 data = subset(df.survivorship.outplantalive.AFRC, species=="MCAP"),
 family = binomial,
 control = glmerControl(optimizer = "nloptwrap"))

## Warning in checkConv(attr(opt, "derivs"), opt$par, ctrl = control$checkConv, :
## Model failed to converge with max|grad| = 0.00232268 (tol = 0.002, component 1)

summary(glm1.survival.AFRC.MCAP)

## Generalized linear mixed model fit by maximum likelihood (Laplace
## Approximation) [glmerMod]
## Family: binomial ( logit )
## Formula: alive_2 ~ scale(exp_day_outplanted) * size + (1 | genotype/pyramid)
## Data: subset(df.survivorship.outplantalive.AFRC, species == "MCAP")
## Control: glmerControl(optimizer = "nloptwrap")
##
## AIC BIC logLik deviance df.resid
## 242.6 272.1 -113.3 226.6 285
##
## Scaled residuals:
## Min 1Q Median 3Q Max
## -2.9608 -0.2768 0.0694 0.3597 3.6588
##
## Random effects:
## Groups Name Variance Std.Dev.
## pyramid:genotype (Intercept) 6.5990 2.5689
## genotype (Intercept) 0.2744 0.5238
## Number of obs: 293, groups: pyramid:genotype, 27; genotype, 3
##
## Fixed effects:
## Estimate Std. Error z value Pr(>|z|)
## (Intercept) 1.86321 1.02817 1.812 0.06996 .
## scale(exp_day_outplanted) 2.08905 1.03539 2.018 0.04363 *
## sizeMedium -0.49912 0.92020 -0.542 0.58754
## sizeSmall -2.71142 0.90270 -3.004 0.00267 **
## scale(exp_day_outplanted):sizeMedium -0.07669 0.91872 -0.083 0.93348
## scale(exp_day_outplanted):sizeSmall 0.45713 0.88996 0.514 0.60749
## ---
## Signif. codes: 0 '***' 0.001 '**' 0.01 '*' 0.05 '.' 0.1 ' ' 1
##
## Correlation of Fixed Effects:
## (Intr) sc(__) sizMdm szSmll s(__):M
## scl(xp_dy_) 0.367
## sizeMedium -0.709 -0.381
## sizeSmall -0.763 -0.449 0.828
## scl(xp__):M -0.362 -0.706 0.493 0.418
## scl(xp__):S -0.392 -0.763 0.418 0.431 0.820
## optimizer (nloptwrap) convergence code: 0 (OK)
## Model failed to converge with max|grad| = 0.00232268 (tol = 0.002, component 1)

# R2
r.squaredGLMM(glm1.survival.AFRC.MCAP)

## Warning: The null model is correct only if all variables used by the original
## model remain unchanged.

## boundary (singular) fit: see ?isSingular

## R2m R2c
## theoretical 0.406705 0.8079494
## delta 0.390516 0.7757888

# Convergence
relgrad <- with(glm1.survival.AFRC.MCAP@optinfo$derivs,solve(Hessian,gradient))
max(abs(relgrad))

## [1] 0.001994722

#VIF
vif(glm1.survival.AFRC.MCAP)

## GVIF Df GVIF^(1/(2*Df))
## scale(exp_day_outplanted) 2.676286 1 1.635936
## size 1.495689 2 1.105886
## scale(exp_day_outplanted):size 2.994428 2 1.315462

Ok, interesting results here. Good model convertence and low variance inflation. At AFRC we see significant effects of both nursery time and size grouping, but no interaction of the two. The longer fragments stay in nursery the higher their survival post-outplanting. Fragments saw a positive effect of being larger, a negative effect of medium size, and a strong negative effect of small fragment size.

plot_model(glm1.survival.AFRC.MCAP, type = "std", show.intercept = TRUE, show.p = TRUE) +
 theme_classic() +
 labs(title = "Estimated effect-size on fragment P(survival) at 367 days")

## Argument 'df_method' is deprecated. Please use 'ci_method' instead.


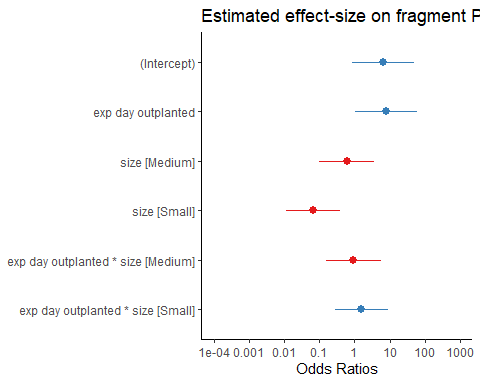


plot_model(glm1.survival.AFRC.MCAP, type = "diag")

## $`pyramid:genotype`

## `geom_smooth()` using formula 'y ~ x'


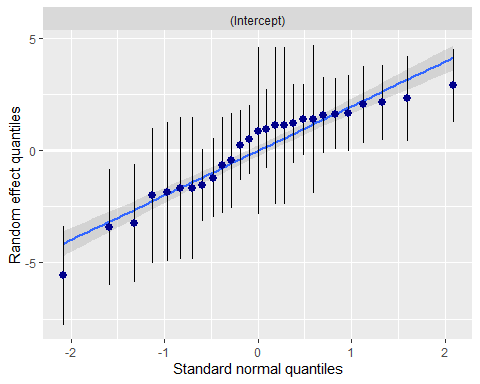


##
## $genotype

## `geom_smooth()` using formula 'y ~ x'


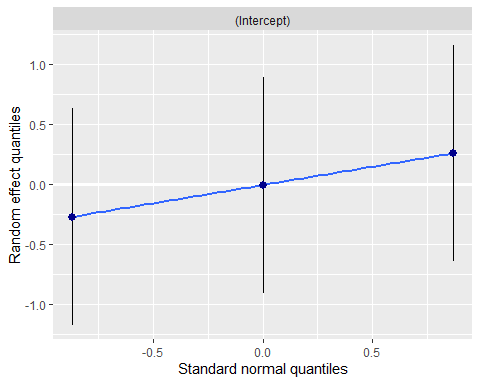


Model diagnostic plots suggest a bit of poor model fit around 0, but it does seem workable. As we saw before almost all variation is attributed to the pyramid, but at AFRC there is more variation among genotypes than was observed at HIMB. The high variability and slight non-normality of pyramid-level variation suggests small-scale, non-random spatio-environmental effects.

**Plotting marginal predicted effects, where non-focal variables are held constant**

plot_model(glm1.survival.AFRC.MCAP, type = "eff", title = "Predicted probability of fragment survival at Day 367", axis.title = c("P(Fragment surviving at Day 367)"))

## Package `effects` is not available, but needed for `ggeffect()`. Either install package `effects`, or use `ggpredict()`. Calling `ggpredict()` now.FALSE

## $exp_day_outplanted


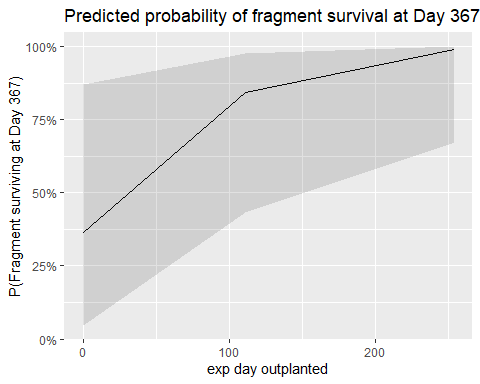


##
## $size


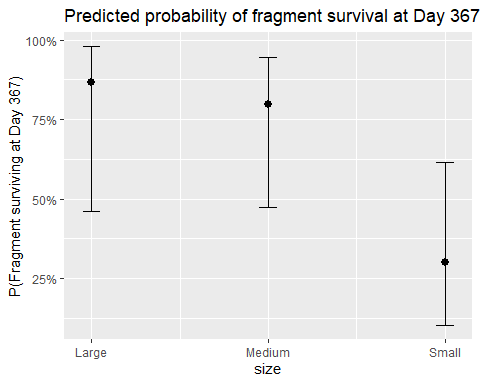


We see here that there is a small difference in the probability of a fragment surviving based on how long it has been in the AFRC nursery, but this is in slight disagreement with the large estimate in the model of nursery time at AFRC. Importantly, the model estimates are conditional, and these are marginal effects plots. The predicted marginal effect of size group is what we observed in the model where small fragments do the worst, medium fragments do better, and large fragments do the best, but all groups are noisy.

**Binomial model of fragment mortality, AFRC PCOMP**

glm1.survival.AFRC.PCOMP <- glmer(alive_2 ~
 scale(exp_day_outplanted)*size + (1|genotype/pyramid),
 data = subset(df.survivorship.outplantalive.AFRC, species=="PCOMP"),
 family = binomial,
 control = glmerControl(optimizer = "nloptwrap"))

## boundary (singular) fit: see ?isSingular

summary(glm1.survival.AFRC.PCOMP)

## Generalized linear mixed model fit by maximum likelihood (Laplace
## Approximation) [glmerMod]
## Family: binomial ( logit )
## Formula: alive_2 ~ scale(exp_day_outplanted) * size + (1 | genotype/pyramid)
## Data: subset(df.survivorship.outplantalive.AFRC, species == "PCOMP")
## Control: glmerControl(optimizer = "nloptwrap")
##
## AIC BIC logLik deviance df.resid
## 286.1 315.7 -135.1 270.1 289
##
## Scaled residuals:
## Min 1Q Median 3Q Max
## -2.6430 -0.3971 -0.1231 0.5123 4.7901
##
## Random effects:
## Groups Name Variance Std.Dev.
## pyramid:genotype (Intercept) 5.612 2.369
## genotype (Intercept) 0.000 0.000
## Number of obs: 297, groups: pyramid:genotype, 27; genotype, 3
##
## Fixed effects:
## Estimate Std. Error z value Pr(>|z|)
## (Intercept) 0.5938 0.7590 0.782 0.434
## scale(exp_day_outplanted) 1.1295 0.7540 1.498 0.134
## sizeMedium -0.9582 0.6789 -1.411 0.158
## sizeSmall -2.8267 0.6884 -4.106 4.02e-05 ***
## scale(exp_day_outplanted):sizeMedium -0.2714 0.6559 -0.414 0.679
## scale(exp_day_outplanted):sizeSmall -0.9467 0.6499 -1.457 0.145
## ---
## Signif. codes: 0 '***' 0.001 '**' 0.01 '*' 0.05 '.' 0.1 ' ' 1
##
## Correlation of Fixed Effects:
## (Intr) sc(__) sizMdm szSmll s(__):M
## scl(xp_dy_) 0.049
## sizeMedium -0.672 -0.093
## sizeSmall -0.679 -0.135 0.812
## scl(xp__):M -0.074 -0.679 0.084 0.100
## scl(xp__):S -0.095 -0.720 0.105 0.157 0.812
## optimizer (nloptwrap) convergence code: 0 (OK)
## boundary (singular) fit: see ?isSingular

# R2
r.squaredGLMM(glm1.survival.AFRC.PCOMP)

## Warning: The null model is correct only if all variables used by the original
## model remain unchanged.

## boundary (singular) fit: see ?isSingular

## R2m R2c
## theoretical 0.1386324 0.6816784
## delta 0.1232406 0.6059944

# Convergence
relgrad <- with(glm1.survival.AFRC.PCOMP@optinfo$derivs,solve(Hessian,gradient))
max(abs(relgrad))

## [1] 0.000273483

#VIF
vif(glm1.survival.AFRC.PCOMP)

## GVIF Df GVIF^(1/(2*Df))
## scale(exp_day_outplanted) 2.200138 1 1.483286
## size 1.035686 2 1.008805
## scale(exp_day_outplanted):size 2.230672 2 1.222106

Convergence warning on this model, but it is still decently converging. VIF is good as well. Whereas for MCAP at AFRC we saw a positive effect of time in nursery, we don’t see a significant effect for PCOMP. In fact the only significant effect here for PCOMP is that smaller fragments has a strongly negative estimated effect on survivorship post-outplanting. This effect is consistent at AFRC across both species.

plot_model(glm1.survival.AFRC.PCOMP, type = "std", show.intercept = TRUE, show.p = TRUE) +
 theme_classic() +
 labs(title = "Estimated effect-size on fragment P(survival) at 367 days")

## boundary (singular) fit: see ?isSingular

## Argument 'df_method' is deprecated. Please use 'ci_method' instead.


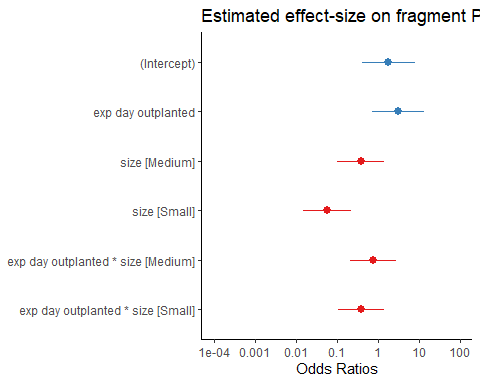


plot_model(glm1.survival.AFRC.PCOMP, type = "diag")

## $`pyramid:genotype`

## `geom_smooth()` using formula 'y ~ x'


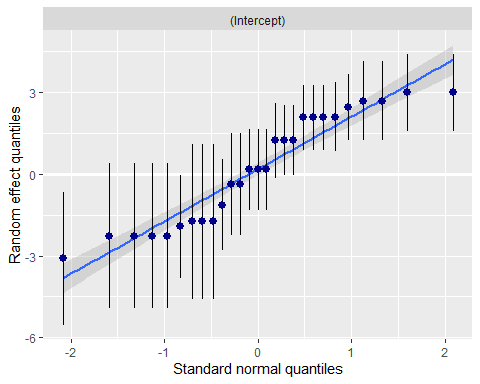


##
## $genotype

## `geom_smooth()` using formula 'y ~ x'


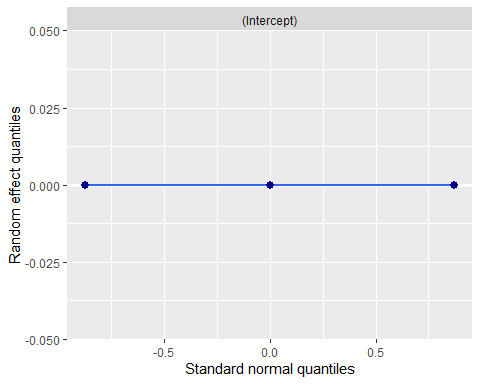


We can see from the above plot that, as before, there is almost no variation across genotypes. Interestingly, there is less variation among genotypes for PCOMP at AFRC than MCAP, whereas this pattern was reversed for HIMB - though in all cases the scale of this variation is tiny. Lastly, as we expect now, almost all of the variation comes down to what pyramid you are on. Luckily, the random effect for pyramid looks quite good against the standard normal quantiles.

**Plotting marginal predicted effects, where non-focal variables are held constant**

plot_model(glm1.survival.AFRC.PCOMP, type = "eff", se=FALSE, title = "Predicted probability of fragment survival at Day 367", axis.title = c("P(Fragment surviving at Day 367)"))

## Package `effects` is not available, but needed for `ggeffect()`. Either install package `effects`, or use `ggpredict()`. Calling `ggpredict()` now.FALSE

## $exp_day_outplanted


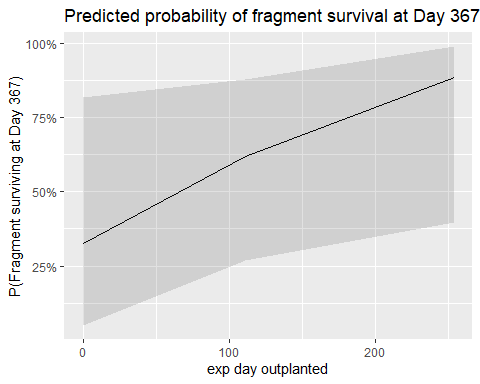


##
## $size


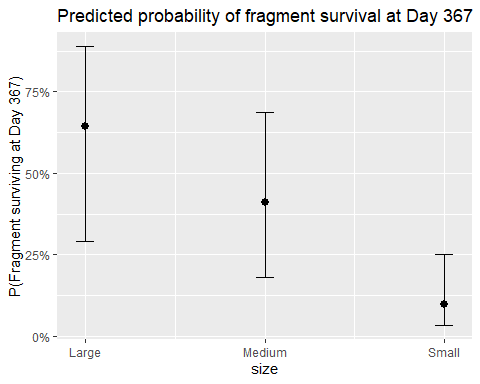


We can see from these marginal plots that for PCOMP at AFRC the number of days held in a nursery has almost no effect at all on fragment survival, whereas fragment size can have large, but highly variable effects on survival post-outplanting. Variability in the probability of surviving is reduced as fragment size decreases.

**Summary of survivorship results:**

- No significant effect of nursery time or size was detected for either species at the HIMB nursery. A marginally insignificant, positive interactive effect between nursery time and medium fragment size was detected for PCOMP at HIMB.
- At AFRC, small fragments of both PCOMP and MCAP were significantly less likely to survive to Day 367, suggesting strong size-dependent effects either in the nursery or at the outplanting location.
- At AFRC, a significant, positive effect of nursery time was found for MCAP but not for PCOMP.
- At either site almost all variation in individual fragment survival came at the pyramid scale and was mostly normally distributed (random).
