# Supplementary material for: Coral micro-fragmentation assays for optimizing active reef restoration efforts
Source: PeerJ. 2022 Jul 18;10:e13653. doi: 10.7717/peerj.13653 (PMC9302430; doi:10.7717/peerj.13653)
Supplement: Supplemental Information 8 — Block assay statistical analyses with Generalized Linear Mixed Models (glmm) examining net growth, and Linear Mixed effects Models (lmm) assessing fragment survivorship of Montipora capitata, and Porites compressa fragments across ten patch reef sites as the fixed effect and genotype as a random effect in Kānéohe Bay, Oʻahu, Hawai'i. [file peerj-10-13653-s008.docx]

Block_assay_statistical_analyses

Ingrid Knapp

11/05/2020

**Set seed**

set.seed(1234)

**Load data**

## site mean median lower upper
## 1 1 127.30170 130.37445 37.676936 216.9265
## 2 2 135.42016 130.63332 34.006174 236.8342
## 3 3 91.05749 93.95791 10.807228 171.3078
## 4 4 80.68854 85.06870 -13.626991 175.0041
## 5 5 106.29663 89.57705 6.275354 206.3179
## 6 6 91.30222 87.45962 -26.430945 209.0354

**Plots_Net_growth**

#Boxplot-a more familiar output, but not in publication
# All sites
#boxplots_all_sites <- ggplot(data=df.g) +
 # geom_boxplot(aes(x=site, y=PercentNetGrowth, fill=site)) +
 # facet_wrap(vars(species)) +
 # labs(x="Site", y="Percent net growth") +
 # theme_classic() +
 # scale_fill_brewer(palette = "Spectral", direction = -1)+
# geom_jitter(aes(x=site, y=PercentNetGrowth))

#boxplots_all_sites

#violin plot

# Flip axes

violin_all_sites_flipped <- ggplot(data = df.g, aes(y = site, x = PercentNetGrowth, fill = site, draw_quantiles)) +
geom_violin(position = position_nudge(x = 0, y = 0), alpha=0.7) +
geom_point(aes(x = PercentNetGrowth), position = position_jitter(width = 0.15), size = 1.4) +
expand_limits(y = 5) +
guides(fill = guide_legend(reverse = TRUE)) +
facet_wrap(vars(species)) +
labs(x="% Net Growth", y="Site") +
scale_color_brewer(palette = "Spectral",direction = -1) +
scale_fill_brewer(palette = "Spectral", direction = -1)+
theme_classic()+
raincloud_theme

violin_all_sites_flipped


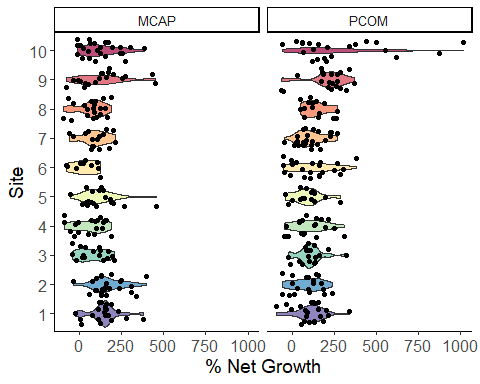


**MCAP_stats_Net_growth**

# Sites seperated (10 sites total)
lm.growth.MCAP <- lmer(PercentNetGrowth ~ site + (1| genotype), data = subset(df.g,species=="MCAP"))
summary(lm.growth.MCAP)

## Linear mixed model fit by REML ['lmerMod']
## Formula: PercentNetGrowth ~ site + (1 | genotype)
## Data: subset(df.g, species == "MCAP")
##
## REML criterion at convergence: 2271.5
##
## Scaled residuals:
## Min 1Q Median 3Q Max
## -2.2858 -0.6679 -0.0124 0.5877 3.4471
##
## Random effects:
## Groups Name Variance Std.Dev.
## genotype (Intercept) 640.9 25.32
## Residual 9071.8 95.25
## Number of obs: 197, groups: genotype, 9
##
## Fixed effects:
## Estimate Std. Error t value
## (Intercept) 140.70 22.12 6.361
## site2 29.28 27.67 1.058
## site3 -70.87 30.41 -2.331
## site4 -79.52 29.97 -2.653
## site5 -25.01 29.92 -0.836
## site6 -108.93 36.47 -2.987
## site7 -60.15 30.86 -1.949
## site8 -71.79 29.58 -2.427
## site9 -11.53 29.46 -0.391
## site10 -16.27 27.67 -0.588
##
## Correlation of Fixed Effects:
## (Intr) site2 site3 site4 site5 site6 site7 site8 site9
## site2 -0.679
## site3 -0.615 0.494
## site4 -0.630 0.502 0.454
## site5 -0.626 0.503 0.456 0.461
## site6 -0.510 0.411 0.372 0.379 0.381
## site7 -0.605 0.485 0.438 0.445 0.448 0.367
## site8 -0.637 0.510 0.460 0.471 0.472 0.388 0.453
## site9 -0.637 0.509 0.461 0.471 0.469 0.385 0.455 0.477
## site10 -0.680 0.543 0.490 0.502 0.501 0.411 0.486 0.511 0.509

Anova(lm.growth.MCAP, type = 3)

## Analysis of Deviance Table (Type III Wald chisquare tests)
##
## Response: PercentNetGrowth
## Chisq Df Pr(>Chisq)
## (Intercept) 40.459 1 2.008e-10 ***
## site 33.986 9 8.983e-05 ***
## ---
## Signif. codes: 0 '***' 0.001 '**' 0.01 '*' 0.05 '.' 0.1 ' ' 1

#normality/HOV
resid_panel(lm.growth.MCAP, plots = "default", type = NA, bins = 30,
 smoother = FALSE, qqline = TRUE, qqbands = FALSE, scale = 1,
 theme = "bw", axis.text.size = 10, title.text.size = 12,
 title.opt = TRUE, nrow = NULL)


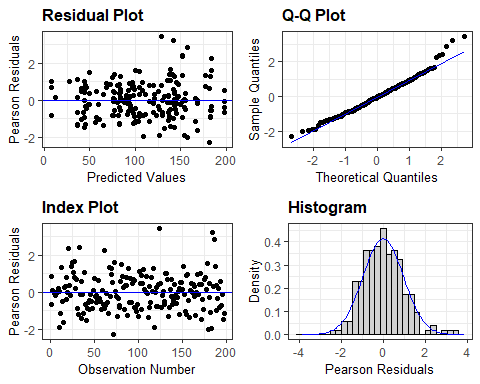
 **Porites_compressa_Net_Growth**

# Sites seperated (10 sites total)
lm.growth.PCOM <- lmer(sqrt(PercentNetGrowth) ~ site + (1| genotype), data = subset(df.g,species=="PCOM"))

## Warning in sqrt(PercentNetGrowth): NaNs produced

## Warning in sqrt(PercentNetGrowth): NaNs produced

## Warning in sqrt(PercentNetGrowth): NaNs produced

summary(lm.growth.PCOM)

## Linear mixed model fit by REML ['lmerMod']
## Formula: sqrt(PercentNetGrowth) ~ site + (1 | genotype)
## Data: subset(df.g, species == "PCOM")
##
## REML criterion at convergence: 951.9
##
## Scaled residuals:
## Min 1Q Median 3Q Max
## -2.6595 -0.5437 -0.0219 0.4929 4.4194
##
## Random effects:
## Groups Name Variance Std.Dev.
## genotype (Intercept) 2.896 1.702
## Residual 14.129 3.759
## Number of obs: 176, groups: genotype, 9
##
## Fixed effects:
## Estimate Std. Error t value
## (Intercept) 11.0055 1.0411 10.571
## site2 -1.2182 1.3069 -0.932
## site3 -0.0462 1.2871 -0.036
## site4 -0.0367 1.2645 -0.029
## site5 -0.5096 1.3935 -0.366
## site6 0.4623 1.2578 0.368
## site7 -1.1221 1.1479 -0.978
## site8 0.3725 1.3026 0.286
## site9 3.8157 1.2238 3.118
## site10 4.6992 1.1940 3.936
##
## Correlation of Fixed Effects:
## (Intr) site2 site3 site4 site5 site6 site7 site8 site9
## site2 -0.555
## site3 -0.572 0.451
## site4 -0.579 0.456 0.472
## site5 -0.523 0.411 0.427 0.433
## site6 -0.572 0.456 0.462 0.471 0.425
## site7 -0.631 0.501 0.512 0.519 0.469 0.519
## site8 -0.554 0.439 0.449 0.455 0.413 0.454 0.499
## site9 -0.593 0.469 0.481 0.489 0.443 0.485 0.533 0.471
## site10 -0.606 0.480 0.492 0.499 0.452 0.495 0.546 0.480 0.513

Anova(lm.growth.PCOM, type = 2)

## Analysis of Deviance Table (Type II Wald chisquare tests)
##
## Response: sqrt(PercentNetGrowth)
## Chisq Df Pr(>Chisq)
## site 49.543 9 1.313e-07 ***
## ---
## Signif. codes: 0 '***' 0.001 '**' 0.01 '*' 0.05 '.' 0.1 ' ' 1

#normality/HOV
resid_panel(lm.growth.PCOM, plots = "default", type = NA, bins = 30,
 smoother = FALSE, qqline = TRUE, qqbands = FALSE, scale = 1,
 theme = "bw", axis.text.size = 10, title.text.size = 12,
 title.opt = TRUE, nrow = NULL)


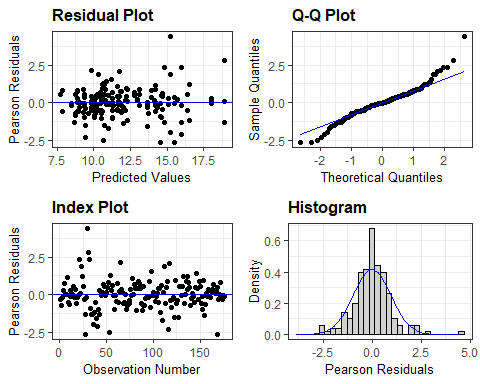


**Survivorship**

**MCAP_Survivorship**

## Generalized linear mixed model fit by maximum likelihood (Laplace
## Approximation) [glmerMod]
## Family: binomial ( logit )
## Formula: alive_2 ~ site + (1 | genotype)
## Data: subset(df.survivorship.outplantalive, species == "MCAP")
## Control: glmerControl(tolPwrss = 0.001, optimizer = "nloptwrap")
##
## AIC BIC logLik deviance df.resid
## 281.6 321.1 -129.8 259.6 256
##
## Scaled residuals:
## Min 1Q Median 3Q Max
## -4.4745 -0.5518 0.3233 0.5951 1.5229
##
## Random effects:
## Groups Name Variance Std.Dev.
## genotype (Intercept) 0.3641 0.6034
## Number of obs: 267, groups: genotype, 9
##
## Fixed effects:
## Estimate Std. Error z value Pr(>|z|)
## (Intercept) 2.0904 0.6599 3.168 0.001536 **
## site2 1.2099 1.1970 1.011 0.312120
## site3 -1.8106 0.7352 -2.463 0.013791 *
## site4 -0.4987 0.8427 -0.592 0.554023
## site5 -0.9201 0.7904 -1.164 0.244410
## site6 -2.6281 0.7672 -3.425 0.000614 ***
## site7 -1.5003 0.7513 -1.997 0.045823 *
## site8 -1.5860 0.7336 -2.162 0.030628 *
## site9 -0.4121 0.8390 -0.491 0.623359
## site10 0.5136 0.9683 0.530 0.595801
## ---
## Signif. codes: 0 '***' 0.001 '**' 0.01 '*' 0.05 '.' 0.1 ' ' 1
##
## Correlation of Fixed Effects:
## (Intr) site2 site3 site4 site5 site6 site7 site8 site9
## site2 -0.495
## site3 -0.816 0.445
## site4 -0.706 0.388 0.635
## site5 -0.754 0.414 0.679 0.589
## site6 -0.788 0.426 0.710 0.610 0.652
## site7 -0.792 0.434 0.712 0.618 0.660 0.686
## site8 -0.815 0.446 0.734 0.635 0.679 0.707 0.712
## site9 -0.707 0.389 0.634 0.552 0.589 0.610 0.620 0.635
## site10 -0.613 0.337 0.551 0.480 0.512 0.529 0.538 0.551 0.481

## Warning: 'r.squaredGLMM' now calculates a revised statistic. See the help page.

## Warning: The null model is correct only if all variables used by the original
## model remain unchanged.

## R2m R2c
## theoretical 0.2521079 0.3266327
## delta 0.1855130 0.2403519

**PCOM_Survivorship**

glm1.survival.PCOM <- glmer(alive_2 ~
 site + (1|genotype),
 data = subset(df.survivorship.outplantalive, species=="PCOM"),
 family = binomial (link = "logit"),
 control = glmerControl(tolPwrss=1e-3, optimizer = "nloptwrap"))

## Warning in checkConv(attr(opt, "derivs"), opt$par, ctrl = control$checkConv, :
## Model failed to converge with max|grad| = 0.02784 (tol = 0.002, component 1)

summary(glm1.survival.PCOM)

## Generalized linear mixed model fit by maximum likelihood (Laplace
## Approximation) [glmerMod]
## Family: binomial ( logit )
## Formula: alive_2 ~ site + (1 | genotype)
## Data: subset(df.survivorship.outplantalive, species == "PCOM")
## Control: glmerControl(tolPwrss = 0.001, optimizer = "nloptwrap")
##
## AIC BIC logLik deviance df.resid
## 216.8 255.0 -97.4 194.8 226
##
## Scaled residuals:
## Min 1Q Median 3Q Max
## -4.1660 0.2778 0.3567 0.4169 0.8544
##
## Random effects:
## Groups Name Variance Std.Dev.
## genotype (Intercept) 0.1236 0.3516
## Number of obs: 237, groups: genotype, 9
##
## Fixed effects:
## Estimate Std. Error z value Pr(>|z|)
## (Intercept) 1.97012 0.62621 3.146 0.00165 **
## site2 -0.04324 0.88561 -0.049 0.96106
## site3 -1.01188 0.76251 -1.327 0.18450
## site4 -0.27744 0.82696 -0.335 0.73725
## site5 -1.21787 0.76952 -1.583 0.11351
## site6 0.39643 0.96397 0.411 0.68089
## site7 -0.28566 0.78939 -0.362 0.71745
## site8 0.72801 1.20144 0.606 0.54455
## site9 0.04981 0.88075 0.057 0.95490
## site10 0.50014 0.97031 0.515 0.60624
## ---
## Signif. codes: 0 '***' 0.001 '**' 0.01 '*' 0.05 '.' 0.1 ' ' 1
##
## Correlation of Fixed Effects:
## (Intr) site2 site3 site4 site5 site6 site7 site8 site9
## site2 -0.683
## site3 -0.792 0.565
## site4 -0.731 0.526 0.602
## site5 -0.784 0.554 0.644 0.593
## site6 -0.626 0.447 0.515 0.477 0.508
## site7 -0.765 0.552 0.631 0.585 0.621 0.499
## site8 -0.502 0.354 0.412 0.379 0.409 0.326 0.398
## site9 -0.687 0.501 0.567 0.528 0.557 0.450 0.553 0.357
## site10 -0.624 0.457 0.515 0.481 0.505 0.409 0.503 0.323 0.457
## optimizer (nloptwrap) convergence code: 0 (OK)
## Model failed to converge with max|grad| = 0.02784 (tol = 0.002, component 1)

r.squaredGLMM(glm1.survival.PCOM)

## Warning: The null model is correct only if all variables used by the original
## model remain unchanged.

## R2m R2c
## theoretical 0.08595059 0.11905087
## delta 0.04017875 0.05565191
