# Supplementary material for: Coral micro-fragmentation assays for optimizing active reef restoration efforts
Source: PeerJ. 2022 Jul 18;10:e13653. doi: 10.7717/peerj.13653 (PMC9302430; doi:10.7717/peerj.13653)
Supplement: Supplemental Information 9 — Pyramid assay summary table with overall percent survivorship (based on the number of fragments) and percent net growth (based on tissue area (cm2)) for Montipora capitata, and Porites compressa for small, medium, and large fragments at both the in-situ and ex-situ nursery sites. Two-dimensional percent net growth was calculated from top-down planar images taken at both nurseries of those fragments which survived till the end, while three-dimensional growth at the in-situ nursery was based on Structure from motion (SfM) models. [file peerj-10-13653-s009.pdf]

	No. of fragments			Tissue area (cm²)				
	**Start**	**End**	**% Survivorship**	**Start**	**End**	**3D End**	**% 2D Net Growth**	**% 3D Net Growth**
Montipora capitata	**594**	**276**	**46%**	**1005.4**	**2058.4**	**NA**	**105%**	**NA**
In-situ nursery	297	123	41%	321.2	697.3	1504.0	117%	368%
Small	189	73	39%	74.2	165.1	365.5	123%	393%
Medium	81	36	44%	152.7	276.9	657.5	81%	331%
Large	27	14	52%	94.3	255.4	481.0	171%	410%
Ex-situ nursery	297	153	52%	684.2	1361.0	NA	99%	NA
Small	189	80	42%	133.8	305.9	NA	129%	NA
Medium	81	54	67%	316.2	666.6	NA	111%	NA
Large	27	19	70%	234.3	388.6	NA	66%	NA
Porites compressa	**594**	**219**	**37%**	874.0	1750.6	NA	100%	NA
In-situ nursery	297	119	40%	373.0	496.6	1068.2	33%	186%
Small	189	58	31%	75.3	93.7	145.9	24%	94%
Medium	81	47	58%	182.6	264.9	623.2	45%	241%
Large	27	14	52%	115.1	137.9	299.1	20%	160%
Ex-situ nursery	297	100	34%	501.0	1254.0	NA	150%	NA
Small	189	46	24%	113.3	200.4	NA	77%	NA
Medium	81	38	47%	209.0	643.0	NA	208%	NA
Large	27	16	59%	178.8	410.7	NA	130%	NA
**Grand Total**	**1188**	**495**	**42%**	**1879.4**	**3809.0**	**NA**	**103%**	**NA**
