# Supplementary material for: Coral micro-fragmentation assays for optimizing active reef restoration efforts
Source: PeerJ. 2022 Jul 18;10:e13653. doi: 10.7717/peerj.13653 (PMC9302430; doi:10.7717/peerj.13653)
Supplement: Supplemental Information 10 — Summary of generalized linear mixed-effects models (glmm) assessing the likelihood of individual fragments surviving to the end of the experiment based on their total time in the ex-situ nursery and their size class (fixed effects), while accounting for variation due to parent colony (genotype) and deployment pyramid within colony (random effects) for (A) Montipora capitata, and (B) Porites compressa fragments at the ex-situ nursery. Overall model performance was evaluated as adjusted marginal versus conditional R2. [file peerj-10-13653-s010.pdf]

| **A)** Ex-situ survivorship | ***Montipora capitata*** | | | |
|---|---|---|---|---|
| **Fixed effects** | **Estimate** | **SE** | **z value** | **Pr (>\|z\|)** |
| Intercept | 1.8640 | 1.0283 | 1.813 | 0.0699 |
| Day outplanted | 2.0903 | 1.0357 | 2.018 | **0.0436** |
| Medium fragments | -0.5002 | 0.9204 | -0.543 | 0.5869 |
| Small fragments | -2.7124 | 0.9030 | -3.004 | **0.0027** |
| Day outplanted: Medium fragments | -0.0782 | 0.9189 | -0.085 | 0.9322 |
| Day outplanted: Small fragments | 0.4557 | 0.8902 | 0.512 | 0.6087 |
| **Random effects** | **Variance** | | **SD** | |
| Pyramid: Genotype | 6.6005 | | 2.5691 | |
| Genotype | 0.2739 | | 0.5234 | |
| **R- squared** | **Marginal** | | **Conditional** | |
| | 0.4066 | | 0.8079 | |
| **AIC** | 242.6 | | | |

| **B)** | ***Porites compressa*** | | | |
|---|---|---|---|---|
| **Fixed effects** | **Estimate** | **SE** | **z value** | **Pr (>\|z\|)** |
| Intercept | 0.5929 | 0.7590 | 0.781 | 0.435 |
| Day outplanted | 1.1289 | 0.7540 | 1.497 | 0.134 |
| Medium fragments | -0.9578 | 0.6788 | -1.411 | 0.158 |
| Small fragments | -2.8259 | 0.6883 | -4.106 | 4.03e-05 |
| Day outplanted: Medium fragments | -0.2717 | 0.6559 | -0.414 | 0.679 |
| Day outplanted: Small fragments | -0.9466 | 0.6498 | -1.457 | 0.145 |
| **Random effects** | **Variance** | | **SD** | |
| Pyramid: Genotype | 5.613e+00 | | 2.3691 | |
| Genotype | 3.162e-08 | | 0.0001 | |
| **R- squared** | **Marginal** | | **Conditional** | |
| | 0.1385 | | 0.6816 | |
| **AIC** | 286.1 | | | |
