# Supplementary material for: Coral micro-fragmentation assays for optimizing active reef restoration efforts
Source: PeerJ. 2022 Jul 18;10:e13653. doi: 10.7717/peerj.13653 (PMC9302430; doi:10.7717/peerj.13653)
Supplement: Supplemental Information 11 — Summary of generalized linear mixed-effects models (glmm) assessing the likelihood of individual fragments surviving to the end of the experiment based on their total time in the in-situ nursery and their size class (fixed effects), while accounting for variation due to parent colony (genotype) and deployment pyramid within colony (random effects) for (A) Montipora capitata, and (B) Porites compressa fragments at the in-situ nursery. Overall model performance was evaluated as adjusted marginal versus conditional R2. [file peerj-10-13653-s011.pdf]

**A)** In-situ survivorship	*Montipora capitata*			
**Fixed effects**	**Estimate**	**SE**	**z value**	**Pr (>|z|)**
Intercept	-0.8625	2.4316	-0.355	0.7230
Day outplanted	0.6489	0.7447	0.871	0.3840
Medium fragments	-0.5031	0.7655	-0.657	0.5110
Small fragments	-0.9665	0.7169	-1.348	0.1780
Day outplanted: Medium fragments	-0.1175	0.8146	-0.144	0.8850
Day outplanted: Small fragments	0.0003	0.7603	0.000	1.000
**Random effects**	**Variance**		**SD**	
Pyramid: Genotype	0.4733		0.688	
Genotype	12.8264		3.581	
**R- squared**	**Marginal**		**Conditional**	
	0.0282		0.8072	
**AIC**	235.1			

**B)**	*Porites compressa*			
**Fixed effects**	**Estimate**	**SE**	**z value**	**Pr (>|z|)**
Intercept	0.4548	0.7670	0.593	0.5532
Day outplanted	-0.1098	0.5674	-0.1930	0.8466
Medium fragments	0.8710	0.6082	1.432	0.1521
Small fragments	-0.8482	0.5369	-1.580	0.1141
Day outplanted: Medium fragments	1.1953	0.6491	1.841	0.0656
Day outplanted: Small fragments	0.2638	0.5459	0.483	0.6289
**Random effects**	**Variance**		**SD**	
Pyramid: Genotype	1.4892		1.2203	
Genotype	0.8591		0.9269	
**R- squared**	**Marginal**		**Conditional**	
	0.1505		0.5043	
**AIC**	272.8			
