# Supplementary material for: Coral micro-fragmentation assays for optimizing active reef restoration efforts
Source: PeerJ. 2022 Jul 18;10:e13653. doi: 10.7717/peerj.13653 (PMC9302430; doi:10.7717/peerj.13653)
Supplement: Supplemental Information 12 — Results of pyramid assay linear mixed effects model (lmm) and type 3 ANOVA output assessing percentage change of net growth (on each pyramid face) relative to fragment size (small (1 cm2), medium (3 cm2) or large (9 cm2)) and nursery residence time (fixed effects), while accounting for variation due to parent colony (genotype) and deployment pyramid within colony (random effects) for (A) Montipora capitata, and (B) Porites compressa fragments at the ex-situ nursery. [file peerj-10-13653-s012.pdf]

**A)** Ex-situ net growth	*Montipora capitata*		
**Fixed effects**	**Estimate**	**SE**	**t value**
Intercept	-38.0312	57.4953	-0.661
Day outplanted	0.5114	0.2574	1.987
Medium fragments	-15.9257	53.1771	-0.299
Small fragments	-60.2118	53.1771	-1.132
Day outplanted: Medium fragments	0.4145	0.3323	1.248
Day outplanted: Small fragments	0.8947	0.3323	2.693
**Random effects**	**Variance**		**SD**
Pyramid: Genotype	3219		56.73
Genotype	4828		69.48
Residual	16112		126.93
**ANOVA**	**Chisq**	**Df**	**Pr(>Chisq)**
Intercept	0.4375	1	0.5083
Day outplanted	3.9494	1	**0.0468**
Group size	1.3769	2	0.5023
Day outplanted: Group size	7.2633	2	**0.0264**

**B)**	*Porites compressa*		
**Fixed effects**	**Estimate**	**SE**	**t value**
Intercept	17.0623	59.6881	0.286
Day outplanted	0.1726	0.3680	0.469
Medium fragments	45.5539	62.9399	0.724
Small fragments	-34.8691	62.9399	-0.554
Day outplanted: Medium fragments	-0.0240	0.3933	-0.061
Day outplanted: Small fragments	-0.0240	0.3933	-0.061
**Random effects**	**Variance**		**SD**
Pyramid: Genotype	16956		130.21
Genotype	282		16.79
Residual	22571		150.24
**ANOVA**	**Chisq**	**Df**	**Pr(>Chisq)**
Intercept	0.0817	1	0.7750
Day outplanted	0.2199	1	0.6391
Group size	1.6423	2	0.4399
Day outplanted: Group size	0.0050	2	0.9975
