# Supplementary material for: Coral micro-fragmentation assays for optimizing active reef restoration efforts
Source: PeerJ. 2022 Jul 18;10:e13653. doi: 10.7717/peerj.13653 (PMC9302430; doi:10.7717/peerj.13653)
Supplement: Supplemental Information 13 — Results of pyramid assay linear mixed effects model (lmm) and type 3 ANOVA output assessing percentage change of net growth (on each pyramid face) relative to fragment size (small (1 cm2), medium (3 cm2) or large (9 cm2)) and nursery residence time (fixed effects), while accounting for variation due to parent colony (genotype) and deployment pyramid within colony (random effects) for (A) Montipora capitata, and (B) Porites compressa fragments at the in-situ nursery. [file peerj-10-13653-s013.pdf]

**A)** In-situ net growth		*Montipora capitata*	
**Fixed effects**	**Estimate**	**SE**	**t value**
Intercept	12.3784	87.3530	0.142
Day outplanted	0.2647	0.2750	0.963
Medium fragments	13.7208	39.7216	0.345
Small fragments	8.62209	39.7216	0.217
Day outplanted: Medium fragments	-0.0217	0.24964	-0.087
Day outplanted: Small fragments	-0.0663	0.2496	-0.266
**Random effects**	**Variance**		**SD**
Pyramid: Genotype	12527		111.93
Genotype	17148		130.95
Residual	8779		93.69
**ANOVA**	**Chisq**	**Df**	**Pr(>Chisq)**
Intercept	0.0201	1	0.8873
Day outplanted	0.9267	1	0.3357
Group size	0.1219	2	0.9409
Day outplanted: Group size	0.0734	2	0.9640

**B)**		*Porites compressa*	
**Fixed effects**	**Estimate**	**SE**	**t value**
Intercept	-40.1829	28.9583	-1.388
Day outplanted	0.0194	0.1514	0.128
Medium fragments	31.1229	26.4796	1.175
Small fragments	20.5874	26.4796	0.777
Day outplanted: Medium fragments	0.1991	0.1664	1.196
Day outplanted: Small fragments	-0.1190	0.1664	-0.715
**Random effects**	**Variance**		**SD**
Pyramid: Genotype	2554.4		50.54
Genotype	775.3		27.84
Residual	3901.2		62.46
**ANOVA**	**Chisq**	**Df**	**Pr(>Chisq)**
Intercept	1.9255	1	0.1653
Day outplanted	0.0164	1	0.8980
Group size	1.4295	2	0.4893
Day outplanted: Group size	3.7292	2	0.1550
