# Supplementary material for: Coral micro-fragmentation assays for optimizing active reef restoration efforts
Source: PeerJ. 2022 Jul 18;10:e13653. doi: 10.7717/peerj.13653 (PMC9302430; doi:10.7717/peerj.13653)
Supplement: Supplemental Information 14 — Text further explaining the difference found between 2D top-down planar image and 3D Structure from Motion (SfM) measurements of coral fragments recorded at the in-situ nursery due to greater arborescent growth here. [file peerj-10-13653-s014.docx]

One interesting finding between the nurseries which arose during the experiment was that although coral micro-fragments typically spread tissue in two dimensions, at the *in-situ* nursery, the coral colonies rapidly grew into complex branching morphologies (see **Figure 3** for a contrast between *in-situ* and *ex-situ* nursery locations). To account for increased surface area due to three dimensionality, we estimated the surface area from 3D SfM models and compared these measurements to those obtained from planar area from scaled digital photographs. Both approaches yielded comparable results according to the generalized linear model (GLM), therefore 2D measurements were modeled throughout. However, in addition to accounting for surface area due to 3D complexity, the SfM segmentation method was significantly faster and required less manual effort. However, the SfM approach may provide a significant time savings. 3D SfM took approximately 10 minutes in the field and 3-5 hours of effort to build and segment the model. In contrast, planer measurements took roughly 5 times longer when the fieldwork (at least 4 digital photographs per module) was combined with at least 25 hours of manually tracing coral tissue cover in imageJ. Therefore, 3D SfM approaches should be considered, particularly when contemplating scaling-up with the desired notion to track a large number of fragments over time which quickly becomes impractical for manual tracing of planar photographs.
