# Supplementary material for: Coral micro-fragmentation assays for optimizing active reef restoration efforts
Source: PeerJ. 2022 Jul 18;10:e13653. doi: 10.7717/peerj.13653 (PMC9302430; doi:10.7717/peerj.13653)
Supplement: Supplemental Information 15 — Block assay survivorship and net growth summary table including the number of Montipora capitata and Porites compressa fragments with any live tissue which survived across the 10 outplanting sites in Kānéohe Bay, Oʻahu, from the start to the end of the experiment, along with percentage survivorship and overall live tissue area cover (cm2) from the start and end with overall percent net growth of those fragments which survived till the end. [file peerj-10-13653-s015.pdf]

	No. of fragments			Tissue area (cm²)		
**Site**	**Start**	**End**	**% Survivorship**	**Start**	**End**	**% Net Growth**
M. capitata	**360**	**197**	**55%**	**832.5**	**1700.7**	**104%**
1	36	22	61%	88.4	209.2	137%
2	36	26	72%	114.1	290.5	155%
3	36	18	50%	84.6	144.8	71%
4	36	19	53%	76.6	119.6	56%
5	36	19	53%	83.9	183.7	119%
6	36	10	28%	34.6	44.2	27%
7	36	17	47%	68.7	124.2	81%
8	36	20	56%	80.0	130.2	63%
9	36	20	56%	89.3	199.0	123%
10	36	26	72%	112.4	255.4	127%
P. compressa	**360**	**201**	**56%**	**699.4**	**1603.7**	**129%**
1	36	22	61%	65.1	133.7	106%
2	36	19	53%	74.4	132.4	78%
3	36	18	50%	72.1	147.9	105%
4	36	21	58%	78.7	154.7	97%
5	36	15	42%	52.3	97.2	86%
6	36	22	61%	72.0	147.0	104%
7	36	26	72%	88.8	173.9	96%
8	36	15	42%	47.7	111.3	133%
9	36	21	58%	74.9	232.7	211%
10	36	22	61%	73.4	273.0	272%
**Grand Total**	**720**	**398**	**55%**	**1531.9**	**3304.4**	**116%**
