# Supplementary material for: Coral micro-fragmentation assays for optimizing active reef restoration efforts
Source: PeerJ. 2022 Jul 18;10:e13653. doi: 10.7717/peerj.13653 (PMC9302430; doi:10.7717/peerj.13653)
Supplement: Supplemental Information 16 — Summary of generalized linear mixed-effects models (GLMM) examining survivorship across 10 patch reef sites as the fixed effect and genotype as a random effect in Kānéohe Bay, Oʻahu, of (A) Montipora capitata, and (B) Porites compressa fragments. The estimates represent the likelihood a coral fragment survived from the time of outplanting to the end of the experiment. Overall model performance was evaluated as adjusted marginal versus conditional R2. [file peerj-10-13653-s016.pdf]

**A)** Site survivorship	*Montipora capitata*			
**Fixed effects**	**Estimate**	**SE**	**z value**	**Pr (>|z|)**
Intercept	2.0906	0.6600	3.168	**0.0015**
Site 2	1.2089	1.1968	1.010	0.3125
Site 3	-1.8109	0.7353	-2.463	**0.0138**
Site 4	-0.4992	0.8428	-0.592	0.5537
Site 5	-0.9207	0.7905	-1.165	0.2441
Site 6	-2.6284	0.7673	-3.425	**0.0006**
Site 7	-1.5006	0.7514	-1.997	**0.0458**
Site 8	-1.5865	0.7337	-2.162	**0.0306**
Site 9	-0.4129	0.8391	-0.492	0.6227
Site 10	0.5123	0.9681	0.529	0.5967
**Random effects**	**Variance**		**SD**	
Genotype	0.364		0.6033	
**R- squared**	**Marginal**		**Conditional**	
	0.2520		0.3265	
**AIC**	281.6			

**B)**	*Porites compressa*			
**Fixed effects**	**Estimate**	**SE**	**z value**	**Pr (>|z|)**
Intercept	1.9688	0.6259	3.146	**0.0017**
Site 2	-0.0459	0.8848	-0.052	0.9586
Site 3	-1.0103	0.7623	-1.325	0.1850
Site 4	-0.2766	0.8267	-0.335	0.7379
Site 5	-1.2160	0.7693	-1.581	0.1140
Site 6	0.3981	0.9639	0.413	0.6796
Site 7	-0.2831	0.7893	-0.359	0.7198
Site 8	0.7282	1.2008	0.606	0.5443
Site 9	0.0548	0.8812	0.062	0.9504
Site 10	0.5025	0.9704	0.518	0.6046
**Random effects**	**Variance**		**SD**	
Genotype	0.1235		0.3514	
**R- squared**	**Marginal**		**Conditional**	
	0.0859		0.1189	
**AIC**	216.8			
