# Supplementary material for: Coral micro-fragmentation assays for optimizing active reef restoration efforts
Source: PeerJ. 2022 Jul 18;10:e13653. doi: 10.7717/peerj.13653 (PMC9302430; doi:10.7717/peerj.13653)
Supplement: Supplemental Information 17 — Results of block assay linear mixed effects model (lmm) and type 3 ANOVA output assessing percentage net growth across 10 patch reef sites in Kānéohe Bay, Oʻahu, of (A) Montipora capitata, and (B) Porites compressa fragments, with genotype as a random effect. [file peerj-10-13653-s017.pdf]

**A)** Site net growth	*Montipora capitata*		
**Fixed effects**	**Estimate**	**SE**	**t value**
Intercept	140.70	22.12	6.361
Site 2	29.28	27.67	1.058
Site 3	-70.87	30.41	-2.331
Site 4	-79.52	29.97	-2.653
Site 5	-25.01	29.92	-0.836
Site 6	-108.93	36.47	-2.987
Site 7	-60.15	30.86	-1.949
Site 8	-71.79	29.58	-2.427
Site 9	-11.53	29.46	-0.391
Site 10	-16.27	27.67	-0.588
**Random effects**	**Variance**		**SD**
Genotype	640.9		25.32
Residual	9071.8		95.25
**ANOVA**	**Chisq**	**Df**	**Pr(>Chisq)**
Site	33.986	9	**8.983e-05**

**B)**	*Porites compressa*		
**Fixed effects**	**Estimate**	**SE**	**t value**
Intercept	11.0055	1.0411	10.571
Site 2	-1.2182	1.3069	-0.932
Site 3	-0.0462	1.2871	-0.036
Site 4	-0.0367	1.2645	-0.029
Site 5	-0.5096	1.3935	-0.366
Site 6	0.4623	1.2578	0.368
Site 7	-1.1221	1.1479	-0.978
Site 8	0.3725	1.3026	0.286
Site 9	3.8157	1.2238	3.118
Site 10	4.6992	1.1940	3.936
**Random effects**	**Variance**		**SD**
Genotype	2.896		1.702
Residual	14.129		3.759
**ANOVA**	**Chisq**	**Df**	**Pr(>Chisq)**
Site	49.543	9	**1.313e-07**
